# A Novel Role for Coilin in Vertebrate Innate Immunity

**DOI:** 10.1096/fj.202403276R

**Published:** 2025-04-25

**Authors:** Douglas M. McLaurin, Sara K. Tucker, Shanzida J. Siddique, Lavanya Challagundla, Yann Gibert, Michael D. Hebert

**Affiliations:** ^1^ Department of Cell and Molecular Biology The University of Mississippi Medical Center Jackson Mississippi USA

## Abstract

Coilin is a protein localized in the nucleus, where it plays a role in the assembly of the Cajal Body and is involved in ribonucleoprotein biogenesis. Our recent research has uncovered new roles for coilin, including its involvement in producing microRNAs and in modifying other proteins through phosphorylation and SUMOylation. We also proposed that coilin could respond to stress signals. In plants, coilin has been shown to help regulate immune genes and activate defense mechanisms, especially in response to stress. In this study, we used two vertebrate models to study coilin function: a human primary foreskin fibroblast cell line deficient in coilin through RNA interference and a newly created zebrafish line with a mutation in the coilin gene generated by CRISPR‐Cas9. Transcriptomic analysis in these two models of coilin deficiency revealed dysregulation of immunity‐related genes in both species. To phenotypically validate the transcriptomic results, we challenged zebrafish coilin mutants with lipopolysaccharide (LPS), which triggers an innate immune response, and identified an attenuated response to LPS in vivo in the zebrafish coilin mutants. Our results support a vital novel function for coilin in vertebrates in regulating the expression of immunity‐related genes. Moreover, these findings could lead to more research on how coilin regulates innate immunity in animals and humans.

## Graphical abstract

In this work, the coilin protein was reduced in human cells, and a novel zebrafish model was followed by treatment with an inducer (lipopolysaccharide) of the innate immunity pathway. RNA sequencing techniques were used to identify differential gene expression and, along with a neutrophil migration assay, demonstrated that coilin promotes a robust innate immune response in vertebrates.

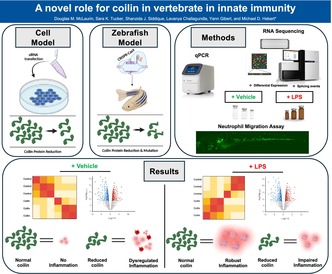

## 1 Introduction

Coilin is a scaffolding protein vital to the assembly of the Cajal body (CB), a subnuclear domain [1, 2, 3, 4]. Coilin self‐associates and binds a host of RNAs and proteins, including Nopp140, to facilitate CB assembly [5, 6, 7, 8, 9]. Other coilin protein binding partners include the survival of motor neuron (SMN) protein, which is crucial to the biogenesis of the small nuclear ribonucleoproteins (snRNPs) [6, 7, 10, 11, 12, 13], and WRAP53, which is responsible for the maturation of telomerase [6, 9, 14]. Through coilin–SMN interactions, snRNPs are trafficked to CBs as part of their biogenesis [7, 12, 15, 16, 17]. SMN dysfunction results in the disease spinal muscular atrophy [18, 19, 20, 21, 22] and coilin mutation has also been linked with defective splicing [23, 24, 25].

Much of coilin's known functions are attributed to its role in the assembly and function of the CB [1, 2, 3, 4, 8, 26, 27, 28, 29]. However, our lab has recently discovered a novel role for coilin as a positive regulator of microRNA (miRNA) biogenesis [30, 31, 32, 33], possibly by the promotion of DGCR8 post‐translation modification [32, 34] or the m^6^A modification of primary miRNAs [35, 36, 37]. Additionally, we have found that coilin regulates NF‐κB signaling through the biogenesis of miR‐517 [30] and the hypoxamir miR‐210 [31], suggesting that human coilin may play a role in the stress response.

In plants, coilin has also been implicated in the stress response [23, 38, 39, 40], including the expression of genes involved in innate immunity and the response to fungal and viral infection [23, 38]. This suggests that coilin's role in the stress response is a conserved function that can be studied across different organisms.

In this study, we sought to examine the role of vertebrate coilin in the innate immune response in human cells and zebrafish embryos through employing transcriptomic analyses. In human cells, we used RNA interference (RNAi) to deplete coilin in the presence or absence of lipopolysaccharide (LPS). These human samples were subjected to RNA sequencing to evaluate differential gene expression and differential alternative splicing events as well as small RNA sequencing to examine differential miRNA gene expression. We also applied CRISPR/Cas9 to engineer coilin mutant zebrafish. These coilin mutants were subjected to LPS injection followed by the analysis of inflammation markers, neutrophil accumulation, and RNA sequencing to assess differential gene expression and alternative splicing events. Our results show that vertebrate coilin plays a significant role in mediating the innate immune response, both in the presence or absence of LPS, by regulating the expression of immune pathways across human cell lines and zebrafish embryos.

## 2 Materials and Methods

### 2.1 Cell Lines, Transfections, and Drug Treatments

Human foreskin fibroblast (primary) cell lines were obtained from the American type culture collection (ATCC). Cells were cultured as previously described [8]. All siRNAs are from Integrated DNA Technologies (Coralville, IA) and used with RNAiMax (Invitrogen, Carlsbad, CA) according to the manufacturer's protocol. Negative control (N), Coilin 2 (I2), coilin A (IA), and WRAP53 (W) were previously described [6, 41]. All siRNA transfections were done for 72 h. LPS (Invitrogen, Carlsbad, CA, USA) treatment was done 24 h prior to harvest at a concentration of 2.0 μg/mL.

### 2.2 HFF RNA Isolation, Sequencing, and Data Analysis

Total RNA was extracted with Tri‐Reagent (Molecular Research Center), according to the manufacturer's instructions and assessed for quality control parameters of minimum concentration and fidelity (i.e., 18S and 28S bands, RIS > 0). Libraries were developed at the University of Mississippi Medical Center's Molecular and Genomics Core Facility (UMMC MGCF) using the Illumina TruSeq mRNA Stranded Library Prep Kit (IDT for Illumina—TruSeq RNA UD Indexes), quantified with a Qubit fluorimeter (Invitrogen), and assessed for quality and size using the QIAxcel Advanced System as done previously [42, 43]. Samples were pooled into a single library (*n* = 24 pooled samples per library) and sequenced using the NextSeq 2000 P3 200 cycles (paired‐end 100 bp) on the Illumina NextSeq2000 platform. The run generated ~1.6 billion reads (%QC30 = 94) and were assessed for quality using FastQC [44]. The raw reads were aligned using the STAR aligner (STAR‐2.7.11a) [45] to the Human reference genome (GRCh38.p14) with corresponding annotation GTF files. The alignment was performed with default parameters, and uniquely mapped reads were retained for downstream analysis. FeatureCounts (2.0.2) [46] was used to generate the count data. The gene‐level count data obtained from FeatureCounts were used for this analysis and were imported into R. Lowly expressed genes, defined as those with low counts across samples, were filtered out (< 10). The remaining count data were then subjected to normalization using the DESeq2 [47] package's built‐in normalization methods, which account for differences in library sizes and biological variability. The normalized count data were used to perform differential expression analysis. A negative binomial distribution model was applied to model the count data, and dispersion estimation was performed for each gene. Differential expression analysis was carried out by fitting the model, taking into consideration the experimental design and conditions of interest. To control for false positives due to multiple hypothesis testing, the resulting *p*‐values were adjusted for multiple testing using methods such as the Benjamini‐Hochberg procedure. Genes with an adjusted *p*‐value at the specified threshold (≤ 0.1) were considered statistically significant. Volcano plots, MA‐plots, heatmaps, and principal components analysis plots based on ‘regularized log’ transformation were used to visualize the results of the differential expression analysis and were generated with R packages ggplot2, EnhancedVolcano, cluster, and heatmap (R ver 4.2.4) [48, 49]. These visualizations aided in highlighting the most significantly upregulated and downregulated genes and patterns across different conditions. The raw and processed data can be accessed from the Gene Expression Omnibus (GEO) accession GSE268257. We utilized the R/Bioconductor package clusterProfiler 4.12.0 [50] to run Gene Ontology (GO) analysis using the significant gene list and a background gene list as input. Subsequently, REVIGO [51] was employed to plot the enriched GO terms against their enrichment *p*‐values, utilizing a semantic similarity measure.

### 2.3 Differential Alternative Splicing Analysis (DAS)

To investigate alternative splicing events, rMATS (replicate Multivariate Analysis of Transcript Splicing) turbo v4.3.0 [52], which is designed to detect and analyze such events from previously aligned bam files, was used to compare the splicing patterns between our conditions of interest. The software efficiently identified differential splicing events, including skipped exons, alternative 5′ and 3′ splice sites, mutually exclusive exons, and retained introns. The results were then statistically analyzed to determine the significance of the observed splicing variations; we used a cutoff parameter of cstat = 0.0001 and an FDR ≤ 0.05 (or 5%).

### 2.4 Small RNA Seq Data Analysis

Samples for small RNASeq were isolated from the previously extracted Total RNA using the Purelink miRNA Isolation Kit per manufacturer's instructions. Sequencing library was prepped using the PerkinElmer NEXTFLEX small RNA‐Seq v3 kit with Unique Dual Indexes (PerkinElmer, Waltham, USA) and sequenced on an Illumina NextSeq2000 sequencer using a P3 50 cycle flowcell (Illumina, San Diego, USA) at the UMMC MGCF. The run generated ~850 million reads (%QC30 = 96). Cutadapt v4.9 [53] was first used to trim the nextflex adapter sequences (TGGAATTCTCGGGTGCCAAGG) from the raw reads and any reads with length < 18 bp and maximum 30 bp per the recommendations from miRBase v22.1 [54]. Quality control statistics both pre‐ and post‐trimming were generated using FastQC and MultiQC [55]. Reads were first aligned against miRbase using Bowtie v.1.2.2 [56]. Unaligned reads were then aligned to the human reference genome (vGRCh38.p14) obtained from the Ensembl database. Further processing of the aligned BAM files was done using samtools [57], and a custom script was then run to combine all the mature miRNA‐based count files into one file. Following the alignment and count aggregation, differential expression analysis methods were consistent with those described in the RNA Seq analysis section. Raw and processed reads have been deposited at NCBI GEO accession GSE271895.

### 2.5 Zebrafish Husbandry

All animal husbandry and experimental techniques were reviewed and approved by the University of Mississippi Medical Center Institutional Animal Care and Use Committee. The zebrafish were handled in accordance with the specified guidelines of the Institutional Animal Care and Use Committee. Embryos were generated using an AB WT strain received from the Zebrafish International Resource Center and raised in embryonic medium at 28.5°C under standard conditions with a 14 h light/10 h dark cycle.

### 2.6 Guide RNA Synthesis

CRISPR/Cas9 mutagenesis was conducted according to a published protocol [58]. Briefly, single guide RNAs (sgRNAs) for *COIL* mutagenesis were designed using the CRISPOR web tool [59]. The 20‐nucleotide gene targeting sequence chosen to generate (ΔN) *COIL* mutants was: 5′‐GAGTGTCGGATGTGTTGGTT‐3′. Applying the two oligo PCR method [60], a scaffold oligo and a gene specific oligo were used to generate a full‐length PCR product containing a T7 binding site, the gene targeting sequence, and sequence to produce an RNA loop required for Cas9 recognition. PCR was set up using *Taq* DNA polymerase (New England Biolabs) according to the manufacturer's protocol and supplementing water in place of template DNA. Thermocycler was run under the following program: 95°C for 30 s; 40 cycles of 95°C for 10 s, 60°C for 10 s, 72°C for 10 s; followed by 72°C for 5 min. The PCR product was purified using the QIAquick PCR purification kit (Qiagen) according to manufacturer's protocol. To generate sgRNA, purified PCR product was used as a template for in vitro transcription using the MEGAscript T7 Transcription Kit (Invitrogen) according to the manufacturer's protocol. In vitro transcribed sgRNA was purified using the RNA Clean & Concentrator kit (ZYMO Research) according to manufacturer's protocol and analyzed on a NanoDrop 2000 spectrophotometer (ThermoFisher).

### 2.7 CRISPR/Cas9 Mutagenesis

For microinjection, a mix containing 500 ng/μL sgRNA, 500 ng/μL TrueCut Cas9 Protein v2 (Invitrogen), and 0.05% phenol red (#P0290 Sigma‐Aldrich) was prepared using sterile water. Prior to spawning, the sgRNA/Cas9 mix was loaded into a pre‐pulled injection needle (#PG52165‐4, World Precision Instruments) and immediately after spawning, embryos were transferred to an agarose gel cast tray and injected with 1 nL of Cas9/sgRNA mix using the microINJECTOR All‐Digital Multi‐pressure System (MINJ‐D, Tritech Research, Los Angeles, CA). Injected embryos were monitored closely during the first three days of growth, and any embryos that showed signs of mortality or abnormal development were removed. At 96 hpf, injected embryos were randomly selected for an initial screening to check the effectiveness of injection by genotyping.

### 2.8 Genotyping

For embryos at 4 dpf, embryos were individually transferred to a clean 1.5 mL centrifuge tube in embryonic medium and stored on ice for at least 30 min to euthanize, according to the AVMA Guidelines for the Euthanasia of Animals. After ice incubation, E3 was aspirated from the 1.5 mL tube and the dried embryo was fast frozen in liquid nitrogen. For adult zebrafish, fish were sedated in 50 mg/L tricaine. Upon sedation and using sterile instruments, a portion of the caudal fin was excised and transferred to a clean 1.5 mL centrifuge tube. After fin excision, the zebrafish was isolated in a separate swim tank for recovery and monitored for signs of distress or ill health for at least 24 h. To extract genomic DNA, samples were immersed in 20 μL of 1× solution of *Taq* DNA polymerase standard *Taq* buffer (New England Biolabs) and boiled for 10 min at 95°C. Samples were briefly centrifuged and then cooled on ice. Proteinase K (#26160, ThermoFisher) was added to the solution at 10 μg/μL, and the sample was then incubated at 37°C for 1 h. Finally, samples were boiled once again for 10 min and cooled on ice. For genotyping, genomic DNA samples were used as a template for PCR using *Taq* DNA polymerase (New England BioLabs) according to the manufacturer's protocol. PCR products were analyzed by gel electrophoresis in 2% agarose gels supplemented with SYBR Safe DNA gel stain (Invitrogen).

### 2.9 RNA Extraction and cDNA Synthesis

Embryonic media was removed from samples until dry. Embryos were then flash frozen in liquid nitrogen. Total RNA was extracted from zebrafish embryos using a motorized tissue grinder (#12‐1413‐61, Fisher Scientific, Hampton, NH, USA) and TRI‐REAGENT (Molecular Research Center, Cincinnati, OH, USA) according to the manufacturer's protocol. Total RNA was next used to generate cDNA via the iScript reverse transcription (RT) super mix (Bio‐Rad Laboratories, Hercules, CA, USA) according to the manufacturer's protocol. Briefly, 1 μg of total RNA, 4 μL of iScript RT super mix, and nuclease‐free water to a reaction volume of 20 μL were applied to cDNA synthesis. Thermal cycler (Eppendorf, Mastercycler X50) settings for cDNA synthesis were 25°C for 5 min, 46°C for 20 min, 95°C for 1 min, and hold at 4°C.

### 2.10 Western Blotting

Adult male zebrafish were euthanized in tricaine. Using a scalpel and forceps, the testes were dissected from the zebrafish and flash frozen in liquid nitrogen. Testis were then lysed in RIPA buffer (50 mM Tris HCl pH 7.6, 150 mM NaCl, 1% NP‐40, 0.25% Na‐Deoxycholate, 1 mM EDTA, 0.1% SDS) plus protease inhibitor cocktail (ThermoFisher Scientific) and pulverized using a motorized tissue grinder. Lysates were sonicated three times with a Fisher Scientific sonic dismembrator (Model 100) for 5 s each using the output setting of 1 and finally centrifuged at 12 000 rpm for 15 min at 4°C. Lysate was run on a precast 10% Mini‐Protean Gel (Bio‐Rad Laboratories, Hercules, CA, USA). Western transfer and detection were conducted as previously described [6]. Primary antibodies include anti‐coilin (#sc‐32 860, Santa Cruz Biotechnology, Dallas, TX) and anti‐beta‐tubulin (#T5201, Sigma Aldrich, St. Louis, MO). Secondary antibodies used were goat anti‐mouse HRP or goat anti‐rabbit HRP. Bands were detected with SuperSignal West Pico Chemiluminescent Substrate (ThermoFisher Scientific) following the manufacturer's suggested protocol. Imaging was done on a ChemiDoc (BioRad) with QuantityOne software. Adjustments to images were made using the transformation settings on QuantityOne software and applied across the entire image.

### 2.11 Quantitative PCR (qPCR)

Reactions were set up with 50 ng cDNA in Brilliant II SYBR Green qPCR master mix (Agilent, Santa Clara, CA, USA) using an Agilent MX3000P qRT‐PCR system. Oligonucleotides used were obtained from Integrated DNA Technologies (Coralville, IA, USA). A complete list of primers can be found in Table 1. Amplification rates, Ct values, and dissociation curve analyses of products were determined using MxPro (version 4.01) software. Relative expression was determined using the 2−ΔΔCT method [61]. GraphPad Prism was used for post hoc statistical analysis and for histogram generation.

**TABLE 1:** List of qPCR primers (5′ to 3′).

Beta‐Actin	F: CTTTGAGCAGGAGATGGGAACCG
	R: GCAACGGAAACGCTCATTGC
Coilin	F: TGGCCACCTCCAGCCTCAAC
	R: TTCAGCAGAAGGCAGGTAGCATT
IL‐1B	F: TGGACTTCGCAGCACAAAATG
	R: GTTCACTTCACGCTCTTGGATG
IL‐6	F: GGCATTTGAAGGGGTCAGGATC
	R: CGCGTTAGACATCTTTCCGTGC
IL‐4	F: CAGCATATACCGGGACTGGAA
	R: GATAATGGCAGCATGCTTTGGT
STAT1	F: GGCGATGATCATCAGCAGAAACC
	R: GTTTGCTCAGCGTCCTGCAC
IFI44a4	F: GATTCCTCAAGTGGTTGTCATGACC
	R: GACACCGATGCTGGCACTAC
ELF3	F: CATTTCCCTCCATGCATGGACC
	R: CGTAGCCGTGGTCACTCTCTG

### 2.12 LPS Microinjection

At 3 dpf, larvae were anesthetized using 0.02% tricaine and loaded onto an agarose gel cast tray. LPS (1 mg/mL, *E. coli* 0111:B4, #L2630, Sigma Aldrich, St. Louis, MO) was injected into the yolk. As a negative control, a group was injected with phosphate‐buffered saline (PBS). Microinjection (MINJ‐D, Tritech Research, Los Angeles, CA) was performed with a volume of 1 nL per larva. At 1 hpi, larvae were screened, and all dead or dying fish were removed. At 5 hpi, larvae were observed once more for dead or dying fish before collection for analysis.

### 2.13 Neutrophil/Macrophage Imaging

After LPS microinjection, 3 dpf larvae were fixed with 4% PFA overnight at 4°C, washed twice with PBST, and washed once with PBS. After that, the larvae were mounted onto a microscope slide using a thin layer of 1% agarose. Mounted larvae were observed and *Z*‐stack imaged by a Zeiss stereomicroscope. After imaging, mounted larvae were immersed in 55°C nanopore water to melt agarose and then transferred to a nuclease‐free tube for genotyping. Prior to genotyping, the neutrophil (red) and macrophage (green) signals were manually and blindly counted, with the *Z*‐plane of every signal carefully examined to avoid miscounting. Only after counting had been completed, genotypes were assigned to the respective fish.

### 2.14 Zebrafish Embryo RNA Isolation, Sequencing and Data Analysis

RNA was extracted using TRIzol Reagent along with Pure Link RNA Mini Kit (Invitrogen) according to manufacturer instructions and assessed for quality control parameters of minimum concentration and fidelity (i.e., 18S and 28S bands, RIS > 9). Libraries were developed at the University of Mississippi Medical Center's Molecular and Genomics Core Facility (UMMC MGCF) using the Illumina TruSeq mRNA Stranded Library Prep Kit (IDT for Illumina—TruSeq RNA UD Indexes), quantified with a Qubit fluorimeter (Invitrogen), and assessed for quality and size using the QIAxcel Advanced System as done previously [42, 43]. Samples were pooled (*n* = 18 pooled samples per library) and sequenced using the NextSeq 2000 P3 200 cycles (paired‐end 100 bp) on the Illumina NextSeq2000 platform. The run generated ~1.3 billion reads (%QC30 = 91.77) and were assessed for quality using FastQC [44].

### 2.15 Differential Gene Expression Analysis (DEG)

The raw sequencing reads were aligned using the SALMON aligner version 1.1.0 [62]. The transcriptome index was built using the Zebrafish reference genome (GRCz11) along with its corresponding annotation GTF files. Quantification was performed in mapping‐based mode using default settings, resulting in estimates of transcript‐level abundances. The gene‐level count data generated by Salmon was utilized for the downstream analysis. The raw count data was imported into R using Tximport (version 1.32.0) to aggregate counts across samples and obtain summarized gene counts for analysis. Genes with low expression, defined as having fewer than 10 counts across samples, were filtered out. The remaining count data was then subjected to normalization using the DESeq2 [47] package's built‐in normalization methods, which account for differences in library sizes and biological variability. The normalized count data was used to perform differential expression analysis. A negative binomial distribution model was applied to model the count data, and dispersion estimation was performed for each gene. Differential expression analysis was carried out by fitting the model, taking into consideration the experimental design and conditions of interest. To control for false positives due to multiple hypothesis testing, the resulting *p*‐values were adjusted for multiple testing using methods such as the Benjamini‐Hochberg procedure. Genes with an adjusted *p*‐value at the specified threshold (*p*adj < 0.1) were considered statistically significant. Volcano plots, MA‐plots, heatmaps, and principal components analysis plots based on ‘variance stabilizing transformation (VST)’ were used to visualize the results of the differential expression analysis and were generated with R packages ggplot2, EnhancedVolcano, and pheatmap (R version 4.4.1) [48, 49].

These visualizations aided in highlighting the most significantly upregulated and downregulated genes and patterns across different conditions. The raw and processed data can be accessed from the Gene Expression Omnibus (GEO) accession GSE273717. We utilized the R/Bioconductor package clusterProfiler 4.12.0 [50] to perform Gene Ontology (GO) enrichment analysis. We obtained a list of significant genes based on differential expression analysis, following that, GO enrichment analysis was conducted using the gseGO function. The gseGO function calculates enrichment scores for Gene Ontology terms (“ont = ‘ALL’”) using gene symbols (“keyType = ‘SYMBOL’”) from the zebrafish genome annotation database (org.Dr.eg.db). We set the number of permutations (nPerm) to 10 000 and applied a significance threshold (*p*valueCutoff = 0.05). Gene set sizes ranged from 1 to 800 genes (minGSSize = 1, maxGSSize = 800), accommodating a wide spectrum of biological processes. The verbose mode was enabled (verbose = TRUE) to monitor the progress of the analysis. Subsequently, REVIGO [51] was employed to plot the enriched GO terms against their enrichment *p*‐values, utilizing a semantic similarity measure.

### 2.16 Differential Alternative Splicing Analysis (DAS)

To explore alternative splicing events, we employed rMATS (replicate Multivariate Analysis of Transcript Splicing) turbo version 4.3.0 [52]. This tool is specifically designed to detect and analyze alternative splicing events using previously aligned BAM files. These BAM files were derived from raw read alignment to the Zebrafish reference genome (GRCz11) and corresponding annotation GTF files using the STAR aligner (STAR‐2.7.11a) [45], utilizing default parameters. rMATS efficiently identifies various types of differential splicing events, such as skipped exons, alternative 5′ and 3′ splice sites, mutually exclusive exons, and retained introns. The results were then statistically analyzed to determine the significance of the observed splicing variations; we used a cutoff parameter of cstat = 0.0001 and an FDR ≤ 0.05 (or 5%). Furthermore, GO enrichment analysis was also performed on genes involved in differential alternative splicing events using the methods described above with the exception that the significance threshold (*p*valueCutoff) was set to 1.

### 2.17 Statistical Analysis

GraphPad Prism was used for all post hoc statistical analyses. Analyses, first, consisted of a Shapiro–Wilk test to determine normality in the collected data from each experiment. Assuming all datasets had a normal distribution and did not violate an assumption for the ANOVA, the data are then subjected to a one‐way ANOVA with a Dunnett's multiple comparison test, comparing only mutant/LPS datasets to WT/PBS datasets. If the collected data have a normal distribution but violate an assumption of the ANOVA, the data are then analyzed by paired *t*‐test (parametric *t*‐test). If the collected data do not have a normal distribution and violate an assumption of the ANOVA, data are analyzed by the Wilcoxon matched‐pairs signed rank test (nonparametric *t*‐test). *p* values recorded from each of the previously named tests are reported in the figure legends.

## 3 Results

### 3.1 Human Coilin Positively Contributes to the Expression of Innate Immunity Genes in HFF Cells

To examine the potential role for human coilin in regulating the immune response, we conducted whole transcriptome sequencing on RNA isolated from human foreskin fibroblast (HFF) cells in which coilin or WRAP53 expression was suppressed via siRNA‐mediated knockdown (KD). Since HFF cells, like many primary cell lines, have few CBs [63], our use of HFF cells will better assess coilin, as opposed to CB function, thereby simplifying data interpretation. Additionally, fibroblasts play an important role in the innate immune response [64]. LPS was chosen as the stressor since coilin interaction with SMN is altered in macrophages treated with LPS [65] and LPS is a known inducer of the NF‐κB pathway [65]. Each knockdown was conducted for 48 h, followed by treatment with DMSO (vehicle) or LPS (2.0 μg/mL) for 24 h, resulting in 72 h KD and 24 h treatment. Tables S1 and S2 contain multiple tabs with the log fold changes and *p* values for each of the comparisons that were conducted (Table S1 contains a contents tab plus 12 additional tabs and Table S2 contains a contents tab plus 7 additional tabs). Significance was assessed at an FDR‐adjusted *p* value of *p* < 0.1. Confirmation of gene suppression is illustrated in the RNAseq results for coilin or WRAP53 showing a 1.8–2.7 log fold reduction across multiple comparisons (Figure S1). Principal component analysis (PCA) plots and volcano plots of individual comparisons can be found in the supplemental figures (Figures S2–S6).

**FIGURE 1:**
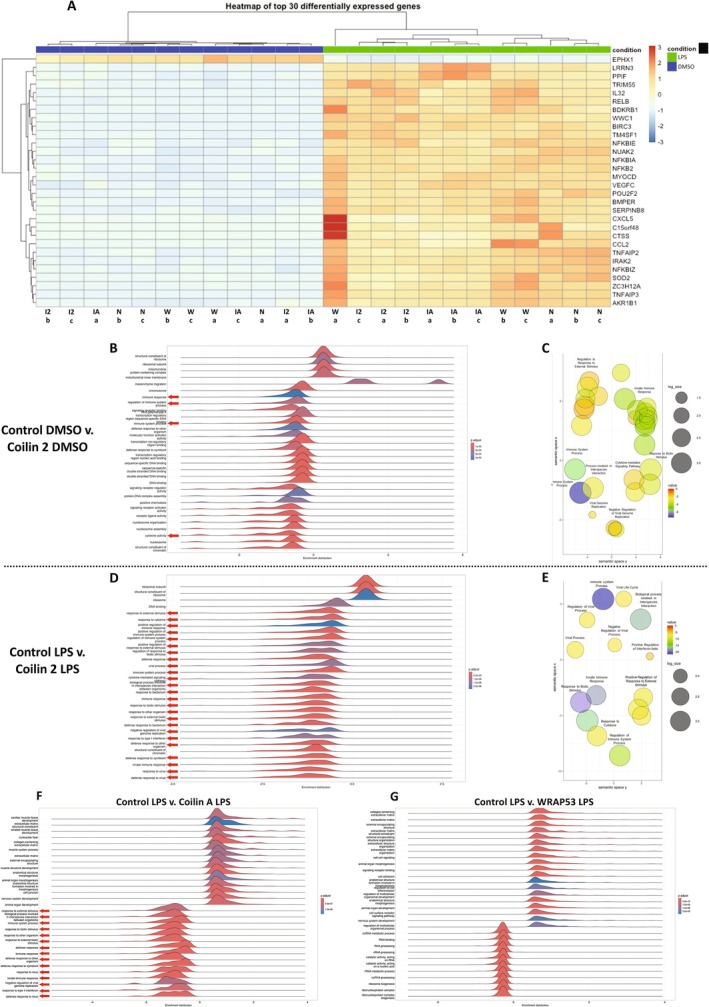
Human coilin positively contributes to the expression of innate immunity genes in HFF cells. (A) Heatmap of the top 30 differentially expressed genes for all KD and treatment (DMSO or LPS) conditions. Control KD indicated as N, coilin 2 KD indicated as I2, coilin A KD indicated as IA, and WRAP53 KD indicated as W. (B) GO analysis for the top differential expressed terms between control versus coilin 2 siRNA treated with DMSO. The red arrows denote terms related to the immune response. Peaks to the left of zero represent decreased expression whereas peaks to the right of zero are induced compared to control. (C) GO cluster analysis of differentially expressed immunity related GO terms for the control versus coilin 2 siRNA treated with DMSO comparison. (D) GO analysis for the top differential terms between control siRNA treated with LPS versus coilin 2 siRNA treated with LPS. The red arrows denote terms related to the innate immune response. (E) GO cluster analysis of differentially expressed genes between control siRNA treated with LPS and coilin 2 siRNA treated with LPS showing prominent clusters for innate immunity. (F) GO analysis for the top differential terms between control siRNA treated with LPS versus coilin A siRNA treated with LPS. The red arrows denote terms related to the innate immune response. (G) GO analysis for the top differential terms between control siRNA treated with LPS versus WRAP53 siRNA treated with LPS.

A heatmap of the top differentially expressed genes (DEGs) shows that numerous immune response‐related genes, such as *SOD2*, *NFKBIZ*, *NFKBIA*, *TNFAIP3*, *CCL2*, and *NFKBIE*, which are regulators or targets of the NF‐κB pathway, are upregulated as a result of LPS treatment (Figure 1A). We found it interesting that amongst both coilin KDs, coilin 2 (I2), or coilin A (IA), there was an attenuation of the induction of these genes (indicated by the reduced intensity of red) in response to LPS treatment compared to that obtained with control or WRAP53 siRNA (Figure 1A, Table S1, tabs 1.5, 1.6, and 1.7). This data suggests that reduced coilin expression under LPS stimulation can impair, but not completely abolish, the immune response. The blunted upregulation of immunity‐related genes upon coilin 2 KD was validated by Western blot analysis of STAT1, SOD2, and IL‐1β (Figure S7A). A typical coilin KD at the protein level is shown in Figure S7B.

To further examine the pathways that may be altered under coilin KD, we then did a gene ontology (GO) enrichment analysis on multiple comparisons to look at clusters of DEGs. Enrichment scores and *p* values are shown for these comparisons in Table S2, tabs 2.1, 2.2, 2.3 and 2.4. Our first comparisons focused on control versus coilin 2 either under DMSO treatment (Figure 1B,C) or LPS treatment (Figure 1D,E). Under both DMSO and LPS treatment, we observed that the enrichment distribution for immunity related GO terms (red arrows) was decreased upon coilin 2 KD (Figure 1B,D), suggesting that coilin 2 KD results in reduced expression of immunity related genes compared to control indiscriminate of a proinflammatory stimulus. Using the reduce + visualize gene ontology (REVIGO) software [51], we clustered and visualized the top immunity related GO terms for control DMSO versus coilin 2 DMSO (Figure 1C) and control LPS versus coilin 2 LPS (Figure 1E). A list of the 46 GO immunity related terms used for the REVIGO analysis is shown in Table S2, tab 2.5. This list consists of terms such as immune system process (GO:0002376), immune response (GO:0006955), and defense response to other organisms (GO:0098542), for example. We extended our analyses to the control versus coilin A (Figure 1F) comparison under LPS treatment. Similar to coilin 2 KD, we found that the enrichment distribution of immunity related GO terms (red arrows) among the control versus coilin A comparison was reduced (Figure 1F). Lastly, we prepared a Venn diagram of the differentially expressed immunity‐related genes in both coilin KD comparisons that are enriched in the GO terms found in Figure 1D,F (Figure S8). Here, we find considerable overlap in the dysregulated genes with 58% and 40% of the genes shared between the coilin A and coilin 2 KDs, respectively (Figure S8). This data suggest that coilin KD mediates a downregulation of genes in these pathways compared to control and this impact is shared across two different coilin siRNAs.

In contrast, the reduction of WRAP53 in the presence of LPS does not dysregulate any of the 46 listed GO terms associated with immunity that were observed under coilin KD (Figure 1G). Instead, the top differentially regulated GO terms upon WRAP53 reduction are associated with non‐coding RNA metabolism or RNP biogenesis consistent with its role as an important telomerase and RNP biogenesis factor. This is an important distinction because coilin and CBs are known to contribute to the biogenesis of RNPs [14, 66, 67, 68, 69, 70]. However, in this HFF cell line, a primary cell line in which < 10% of cells have CBs (our observations) [63], the GO analysis shows that coilin contributes more to the innate immune response than RNP biogenesis, especially in response to LPS stress.

### 3.2 Coilin Contributes to the Innate Immune Response Independent of Splicing Activity in HFF Cells

**FIGURE 2:**
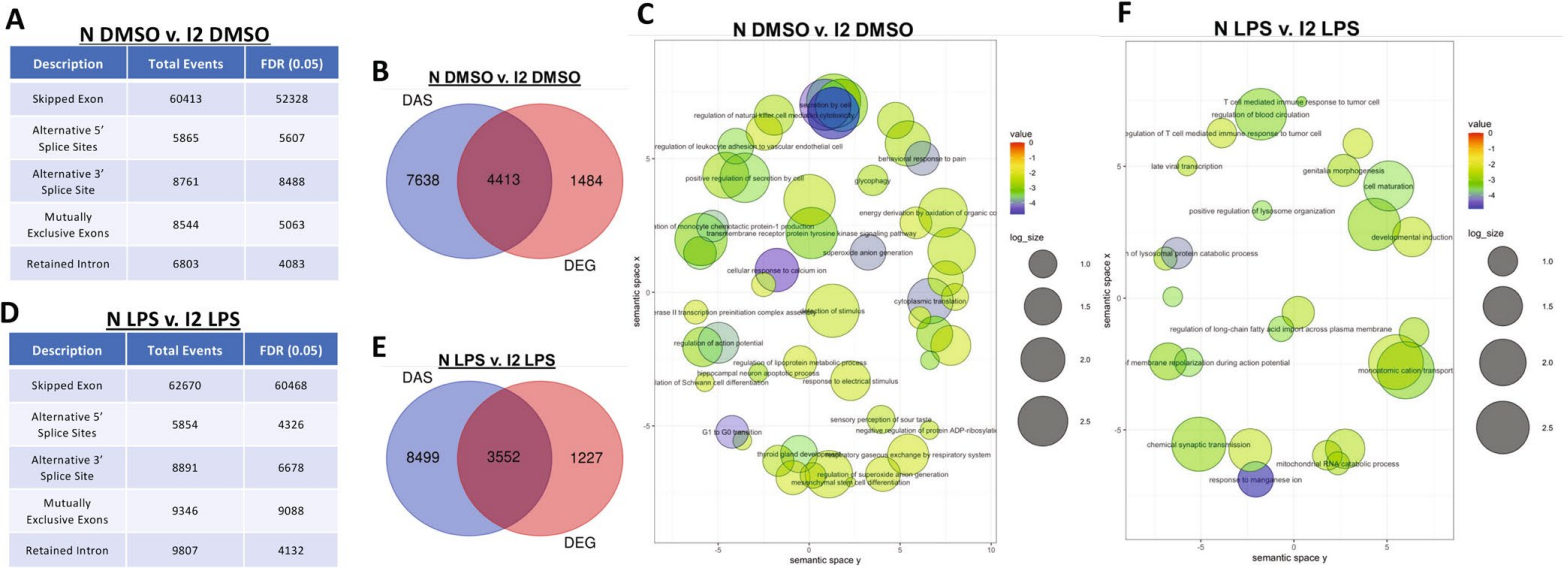
Coilin contributes to the innate immune response independent of splicing activity in HFF cells. (A) rMATS analysis for the control (N) versus coilin 2 (I2) KD treated with DMSO comparison. Five differentially alternatively spliced events were calculated with a false discovery rate (FDR) of 0.05. Compared to control (N), coilin (I2) KD increases all DAS classes. (B) An overlay of DAS genes with DEG genes for DMSO treated control (N) versus coilin (I2) KD. (C) DAS GO cluster analysis showing the top differentially expressed terms for the DMSO treated control (N) versus coilin (I2) KD comparison. (D) rMATS analysis for the control (N) versus coilin 2 (I2) KD treated with LPS comparison. Coilin (I2) KD increases all DAS classes compared to control (N). (E) An overlay of DAS genes with DEG genes for LPS treated control (N) versus coilin (I2) KD comparison. (F) DAS GO cluster analysis showing the top differentially expressed terms for the LPS treated control (N) versus coilin (I2) KD comparison.

Next, we wanted to examine the influence of impaired splicing activity, as a result of coilin reduction, on the innate immune response. For this analysis, we examined five different alternative splicing events using the replicate multivariate analysis of transcript splicing (rMATS) program [52]. The number of differentially alternatively spliced (DAS) events for all five categories increased upon coilin KD compared to control in both DMSO and LPS treatments (Figure 2A,D). These findings show that coilin contributes to the robustness of the splicing machinery even in cell lines lacking many CBs. An overlay of the dysregulated DAS and DEG genes for the control versus coilin 2 DMSO comparison (N DMSO vs. I2 DMSO) shows that 4413 genes are shared (Figure 2B). A similar percentage of shared genes is observed between DAS and DEG genes for the control versus coilin 2 LPS comparison (N LPS vs. I2 LPS, Figure 2E). The large overlap between DAS and DEG genes suggests that DAS may contribute to the innate immunity related differentially expressed genes observed upon coilin KD.

To test this possibility, GO analysis was conducted on the DAS genes for the control versus coilin 2 DMSO and control versus coilin 2 LPS comparisons. Enrichment scores and *p* values are found in Table S2, tabs 2.6 and 2.7. The top differential GO terms on DAS genes for control versus coilin 2 DMSO or LPS comparisons (Figure 2C,F) did not include any of the top immunity‐related GO terms that were recovered in the DEGs upon coilin KD compared to control siRNA (Figure 1C,E, Table S2, tab 2.5). Hence, the immunity‐related DEGs observed with coilin KD, especially in the presence of LPS stress, are not the result of differential alternative splicing events.

### 3.3 Coilin KD Dysregulates MicroRNAs Involved in Innate Immunity

**FIGURE 3:**
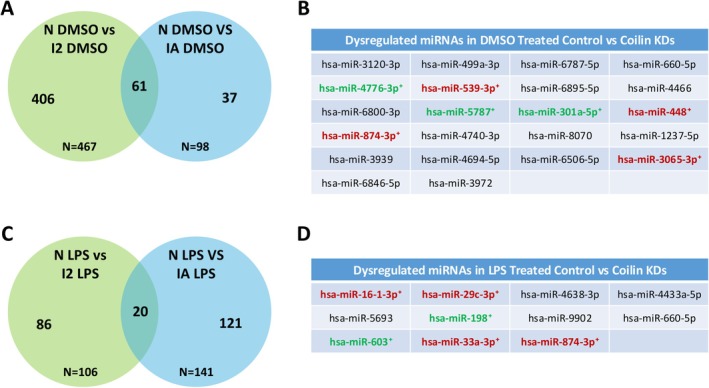
Coilin reduction alters the expression of miRNAs that contribute to the innate immune response. Small RNA seq analysis was conducted and miRNAs significantly dysregulated (*p* ≤ 0.1) in both coilin 2 and coilin A siRNA conditions compared to control (N) siRNA, treated with DMSO are shown. (A, C) Venn diagram showing the number of differentially expressed miRNA genes across coilin 2 (I2) and coilin A (IA) KDs under DMSO or LPS treatment compared to control (N) KD. (B, D) Tables of subsets of miRNAs found to be dysregulated under both coilin I2 and coilin IA KDs with DMSO or LPS treatment compared to control (N) KD. Immunity‐related miRNAs that are upregulated (green) or downregulated (red) in both coilin KD conditions compared to control siRNA are indicated by plus sign (+).

Since our lab has found that coilin knockdown suppresses the biogenesis of several miRNAs, including a regulator of NF‐κB signaling [30, 31, 32, 33], we next evaluated the extent of miRNA dysregulation upon coilin KD in the presence or absence of LPS stress. Log fold changes and *p* values for these comparisons are shown in Table S1, tabs 1.8, 1.9, 1.10, and 1.11. We found that 467 miRNAs were significantly dysregulated in the DMSO control (N) versus coilin 2 (I2) comparison (Table S1, tab 1.8), while 98 were dysregulated in the DMSO control versus coilin A (IA) comparison (Figure 3A, Table S1, tab 1.9). Among these dysregulated miRNAs, 61 were shared in both conditions (Figure 3A, Table S1, tab 1.12). In the LPS comparisons, we observed 106 and 141 significantly dysregulated miRNAs in the coilin 2 and A comparisons, respectively (Figure 3C, Table S1, tabs 1.10 and 1.11).

Some of the significantly dysregulated miRNAs found in both coilin 2 and coilin A siRNA treated cells compared to control siRNA exposed to DMSO (Figure 3B) or LPS (Figure 3D) are shown. Of the 22 miRNA‐subset observed under DMSO comparisons and the 11 miRNAs in the LPS comparisons, seven and six have been found to play a role in innate immunity, respectively (Figure 3B,D, + symbol) [71, 72, 73, 74, 75, 76, 77, 78, 79, 80, 81, 82]. Green colored immunity‐related miRNAs are significantly upregulated in both coilin KD conditions compared to control siRNA, whereas red colored immunity‐related miRNAs are significantly downregulated in both KD conditions compared to control siRNA. Interestingly, miR‐874‐3p was found to be downregulated under all four comparisons (Figure 3B,D), implying a direct correlation between the expression of this miRNA and coilin. This miRNA is thought to be a potent regulator of signaling pathways such as the Wnt/β‐Catenin, Hippo, PI3K/AKT, JAK/STAT, and Hedgehog pathways [77], so its downregulation would likely impair the activity of these pathways under immune system stimulation.

Collectively, these results show that, in addition to differentially expressed and alternatively processed mRNAs, coilin KD with DMSO or LPS stress also dysregulates miRNA biogenesis in HFF cells. As several of these miRNAs are implicated in the immune response, the impact of coilin on miRNA biogenesis likely contributes to the altered gene expression of immunity‐related genes upon coilin reduction.

### 3.4 Generation of Coilin N‐Terminal Mutant Hypomorphs in Zebrafish Using CRISPR/Cas9

**FIGURE 4:**
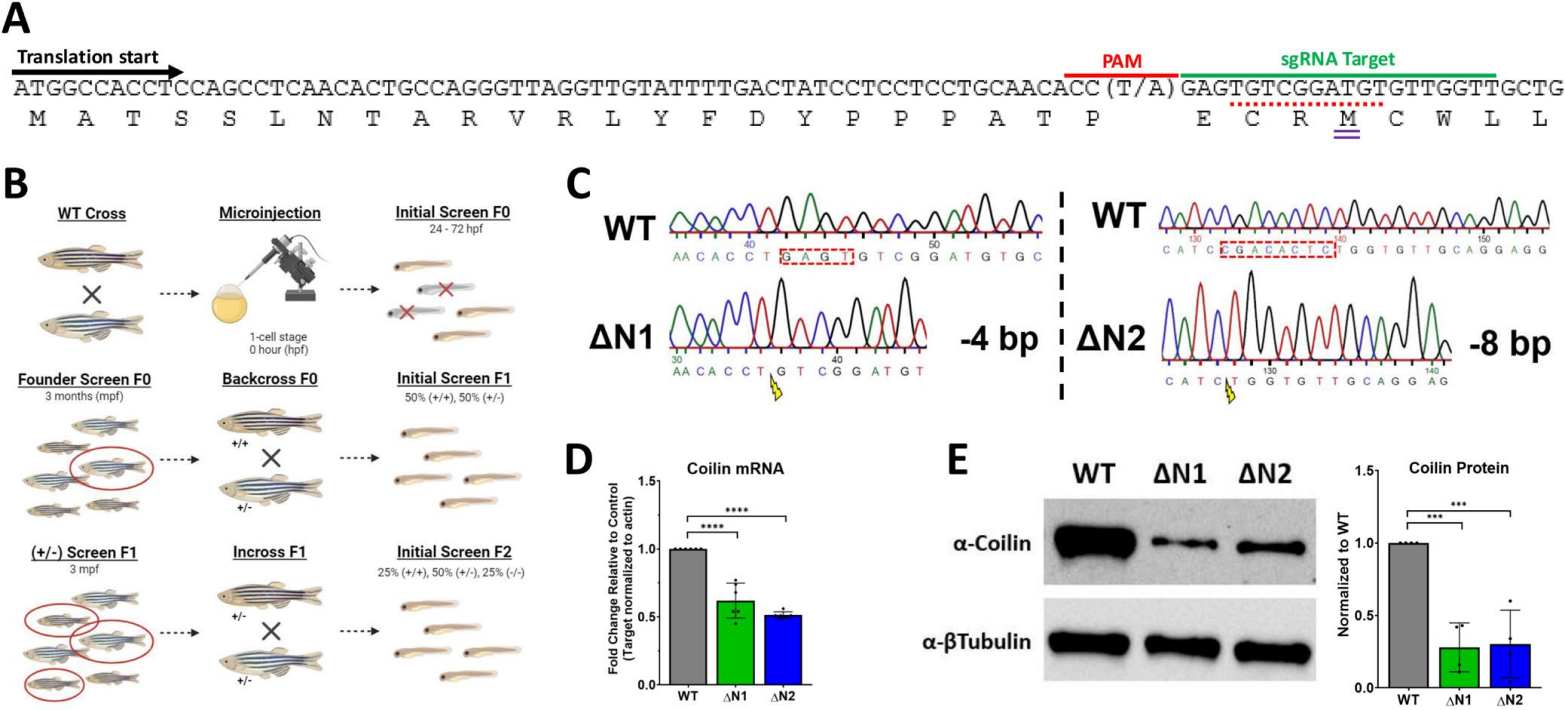
Generation of coilin N‐terminal (ΔN) mutants in zebrafish using CRISPR/Cas9. (A) Schematic of coilin's DNA and amino acid sequences showing the typical translational start site (black arrow), the PAM and sgRNA target sequences used to engineer CRISPR/Cas9 mutagenesis (red and green bars), and the alternative translation start (purple double bar) site lacking a Kozak sequence (red dash). The location of a SNP (A/T) corresponding to the N base of the NGG PAM sequence is shown. (B) Illustration of the development of homozygous *COIL* ΔN mutants. (C) Chromatogram illustrating DNA sequencing results which display the four base pair deletion characterizing the ΔN1 mutation (forward direction) and the eight‐base pair deletion charactering the ΔN2 mutation (reverse direction). The 4 base pair deletion strain has a T at the SNP position and the 8 base pair deletion stain has an A (T when using reverse primer) at this position. Red dash boxes are indicating the deleted sequences and lighting bolts are indicating the site of double‐strand break. (D) Histogram displaying the qPCR results of coilin mRNA expression in *COIL* ΔN1 and ΔN2 fish relative to wild‐type (WT) zebrafish. Data represents 3 biological replicates with 2 technical repeats for a total *N* = 6. Error bars represent SD and black dots represent individual data points. ****p < 0.0001 for comparisons to WT. (E) Western blot visualizing coilin protein expression in the testis of *COIL* ΔN1 and ΔN2 fish relative to WT zebrafish and quantification (histogram). ***p < 0.001.

We next turned our attention toward mutating coilin in a vertebrate model, zebrafish. Because a previous study using coilin reduction in zebrafish with morpholinos resulted in embryonic lethality at 24 h post fertilization (hpf) [70], we generated coilin hypomorphs in order to generate a stable zebrafish line with reduced coilin levels. This was accomplished by using a guide RNA in the CRISPR/Cas9 complex that targets the *coil* gene downstream of the initiator methionine but before a methionine at position 27 (Figure 4A). This guide RNA (5′‐GAGTGTCGGATGTGTTGGTT‐3′) was selected with the help of CRISPOR, an online program that helps design and evaluate guide sequences for CRISPR/Cas9 [59]. We note that the PAM sequence, 5′‐NGG‐3′ on the non‐target strand, contains a single nucleotide polymorphism (A/T) in the corresponding N position of the NGG PAM sequence. To generate *COIL* homozygous mutants, we crossed AB strain wild‐type (WT) zebrafish and injected fertilized embryos at the 1‐cell stage (Figure 4B). Through successive genotyping and crossing across the F0 and F1 generations, we generated homozygous *COIL* mutants in the F2 generation (Figure 4B). Two deletion lines were created using this guide RNA, one with a 4 base pair deletion and another with an 8 base pair deletion (Figure 4C). The 4 base pair deletion strain is homozygous for a T at the SNP position, whereas the 8 base pair deletion stain is homozygous for an A (T when using reverse primer) at this position. *In silico* analysis of these mutations predicted that translation would yield a product with 23 amino acids (aa) of native coilin sequence followed by 9 and 37 aa of non‐native sequence before reaching a stop codon for the −4 and −8 bp deletions, respectively (Figure S9). However, if the ribosome begins translation at the methionine at position 27, this will result in a coilin protein consisting of aa 27–532 (Figure 4A) resulting in an N‐terminal deletion. Hence, we named the mutants ΔN1 (−4 bp deletion) and ΔN2 (−8 bp deletion) (Figure 4C). It is noted that alternative translation initiation is not uncommon when conducting CRISPR/Cas9 mutagenesis [83]. The lack of aa 1–26 may disrupt coilin's ability to self‐associate [4, 7, 84, 85, 86, 87] or interact with Nopp140 [6, 9, 14], both of which may inhibit CB formation. Because M27 lacks a Kozak box (Figure 4A), we suspected that translation of the mRNA emanating from the ΔN1and ΔN2 mutated genes would not be efficient and coilin protein levels would be reduced. To first evaluate if the mutations present in ΔN1 and ΔN2 lines alter coilin mRNA expression, we excised the dorsal fin of adult WT, ΔN1, and ΔN2 zebrafish and extracted total RNA followed by cDNA synthesis. We next performed qPCR and observed an approximate 40% and 50% reduction of coilin mRNA in ΔN1 and ΔN2 mutants, respectively (Figure 4D). We also assessed the protein level of coilin in these mutants by extracting protein lysate from the testis of adult WT, ΔN1, and ΔN2 zebrafish. These lysates were subject to Western blot analysis using an antibody that detects epitopes in the C‐terminus of zebrafish coilin and observed an ~70% reduction in coilin protein levels for both mutants (Figure 4E). As predicted, the reduced amount of coilin protein present in ΔN1and ΔN2 zebrafish is likely the result of the inefficient translation of the mutated mRNA with the ribosome using M27, which lacks a Kozak sequence, as the initiator methionine. The coilin hypomorph lines, ΔN1 and ΔN2, therefore, are consistent with the production of a N‐terminal deletion of coilin encoding aa 27–532. We note that the lack of the first 26 aa of coilin does not obviously change the mobility of coilin compared to WT as detected on a 7.5% polyacrylamide gel (Figure 4E).

### 3.5 Coilin N‐Terminal Mutant Hypomorphs in Zebrafish Do Not Alter Cajal Body Formation, Viability, Morphology, or Reproductivity

Coilin KO models have been obtained in animals for fly [88] and mouse [89]. No obvious phenotype upon coilin KO was observed in fly besides the lack of canonical CBs. The coilin KO mouse, which unfortunately has been lost, had a semi‐lethal phenotype in that there were fewer homozygous null pups born than expected in the most inbred lines. No such decrease was observed in outbred lines [89]. Null pups died late in gestation, and both males and females contributed to the reduction in reproductive fitness [90]. Apart from the fewer null pups born than expected in inbred lines, no other phenotypes for the coilin KO mouse were noted except for the lack of canonical CBs. Coilin reduction by morpholinos in zebrafish results in embryonic lethality at 24 hpf [70]. No such lethality is observed in the coilin N‐terminal mutant hypomorphs we have generated here. Additionally, we do not detect any obvious morphological or reproductive alterations in the coilin N‐terminal mutant hypomorphs lines compared to WT. However, since coilin protein generated from ΔN1and ΔN2 zebrafish is predicted to lack the N‐terminal 26 aa, which may impact CB formation, we next evaluated if CBs were present in these lines. For this analysis, immunofluorescence (IF) was conducted on 48 hpf to detect the core snRNP protein SmB and the 2,2,7‐trimethylated guanosine (m_3_G/TMG) cap found on U snRNAs (except for U6 snRNA), both of which are enriched in CBs. CBs could be detected in WT embryos, heterozygous ΔN1 (+/−) mutants, and homozygous ΔN1 (−/−) mutants (Figure S10). These findings show that the N‐terminal coilin mutant generated by ΔN1 does not abolish canonical CB formation.

### 3.6 Coilin N‐Terminal Mutant Hypomorph Impairs Immunity Pathways

**FIGURE 5:**
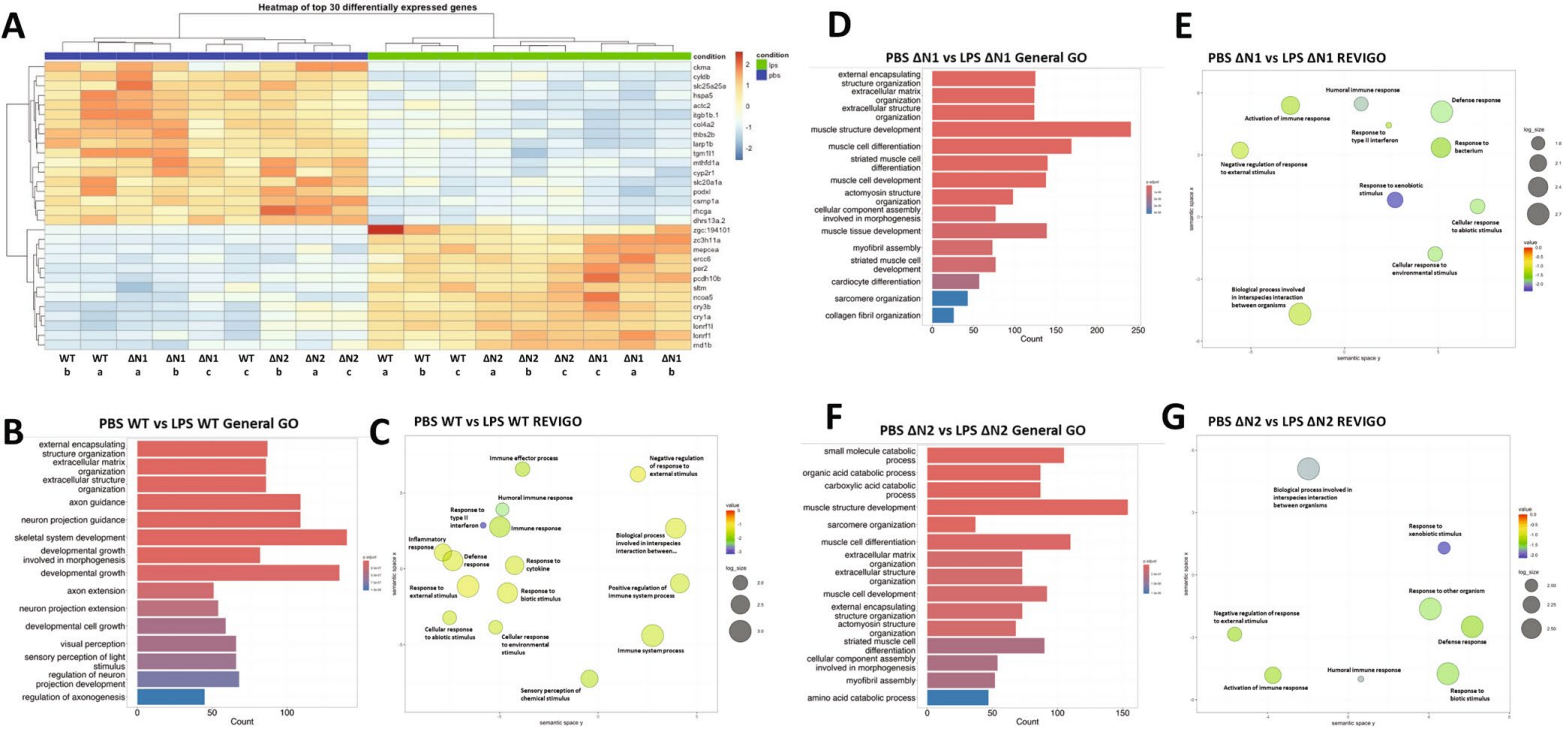
Coilin N‐terminal mutation impairs the activation of immunity‐related pathways. (A) Heatmap of the top 30 differentially expressed genes for WT and coilin mutant (ΔN1 and ΔN2) embryos injected with PBS (blue horizontal bar) or LPS (green horizontal bar). (B, D, F) GO enrichment analysis illustrating the top general pathways altered under PBS versus LPS comparisons in WT and mutants. (C, E, G) REVIGO analysis illustrating the top immunity‐related pathways altered under PBS versus LPS comparisons in WT and mutants.

For a comparative analysis between HFFs and our coilin‐deficient zebrafish mutants, we also subjected PBS or LPS injected embryos to mRNA sequencing. Tables S3 and S4 contain multiple tabs with the log fold changes and *p* values for each of the comparisons that were conducted (Table S3 contains a contents tab plus 8 additional tabs and Table S4 contains a contents tab plus 11 additional tabs). When ranked by the statistical significance (FDR adjusted *p* < 0.1) of standardized expression, we observed that multiple circadian rhythm‐related genes (*Per2*, Ensembl: ENSDARG00000034503; *Cry1a*, ENSDARG00000045768; *Cry3b*, ENSDARG00000091131) are significantly upregulated in the presence of LPS with or without coilin mutation, as well as *Irf1b* (ENSDARG00000043249), a well‐established immune response gene [91, 92] (Figure 5A, Figure S11, Table S3.1). Through targeted PBS versus LPS comparisons (Table S3, tabs 3.2, 3.3, 3.4, 3.5, 3.6, 3.7, and 3.8), we found that *Irf1b* remained amongst the top differentially expressed genes in the WT background, but was absent in the top differentially expressed genes in both coilin mutant backgrounds (Figures S12–S14). This contrasts with the circadian rhythm gene *Per2*, which is present in both coilin mutant comparisons, and *Cry1a* and *Cry3b*, which are present in the ΔN1 background (Figures S12–S14).

Next, we analyzed the DEGs collected from PBS versus LPS comparisons amongst WT and ΔN mutants (Table S3) for GO analysis (Figure 5B–G, Table S4). Our initial analyses found numerous top general pathways being impacted by LPS injection (Table S4, tabs 4.1, 4.2, and 4.3). Several of these pathways are shared between WT and coilin mutants, such as extracellular matrix organization and extracellular structure organization. Interestingly, numerous pathways related to muscle cell differentiation and development were shared between the ΔN mutants but were absent in the WT background. Focusing on immunity‐related terms (Table S4, tab 4.8), we conducted another GO and REVIGO analysis and found a decrease in the activation of immunity‐related pathways as a result of LPS injection in ΔN1 and ΔN2 backgrounds compared to WT (Figure 5C,E,G, Table S4, tabs 4.1, 4.2, and 4.3).

### 3.7 The ΔN1 and ΔN2 Coilin N‐Terminal Mutant Hypomorphs Similarly Influence Gene Expression

**FIGURE 6:**
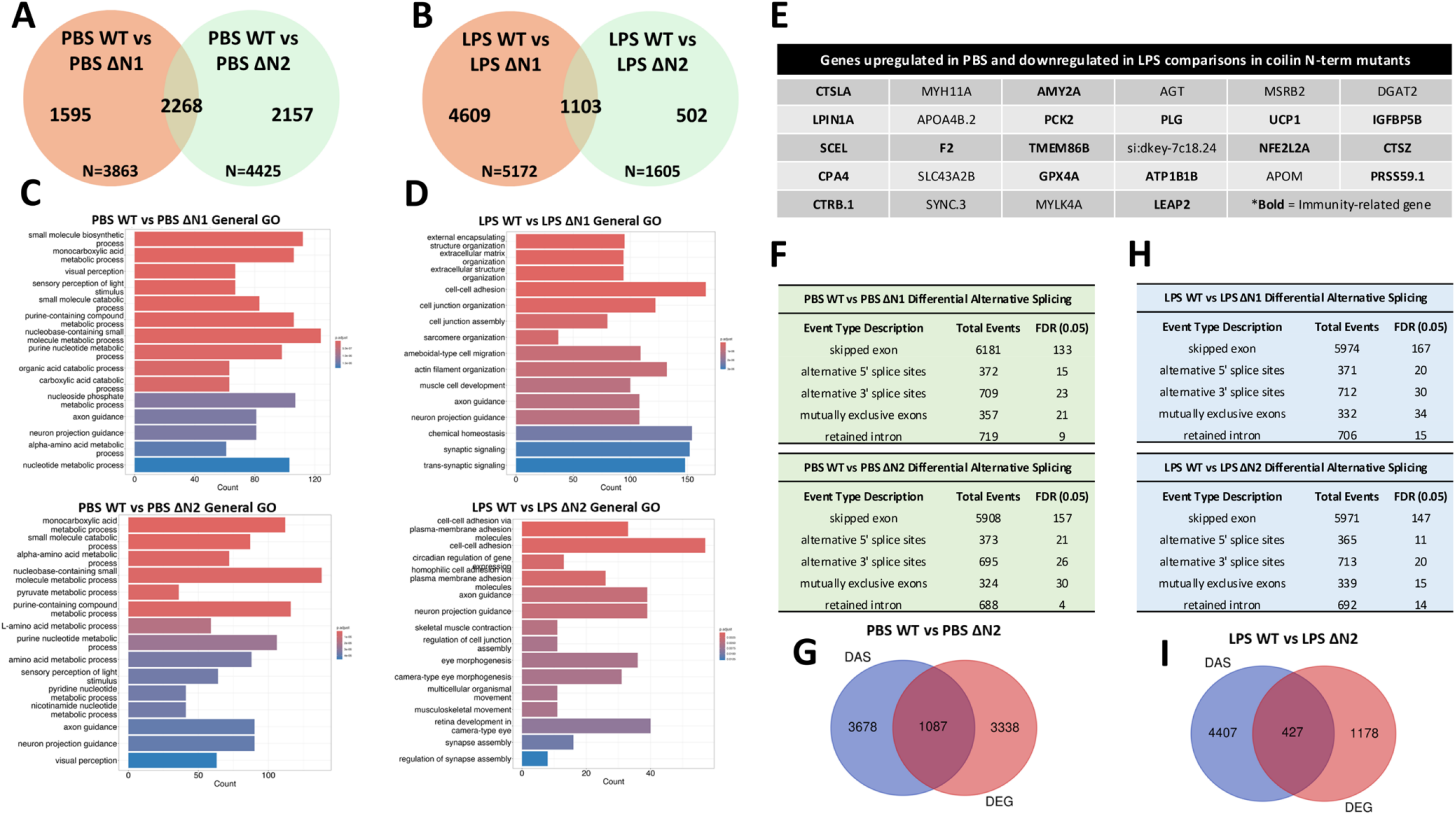
Coilin N‐terminal mutations similarly influence gene expression and predominately act independent of splicing events. (A, B) Venn diagrams illustrating the overlap in genes differentially expressed between ΔN1 and ΔN2 with WT upon PBS or LPS injection. (C, D) GO enrichment analysis illustrating the top general pathways altered under WT versus ΔN mutant comparisons under PBS or LPS injection. (E) Table highlighting a subset of genes found to be upregulated under PBS injection and downregulated under LPS injection in both ΔN mutants compared to WT. Boldened terms highlight immunity‐related genes. (F, H) rMATS analysis for the WT versus ΔN mutant comparisons under PBS or LPS injection. (G, I) Venn diagram showing the number of genes shared across DAS events and differential gene expression in the WT versus ΔN2 comparison under PBS or LPS injection.

We next examined the comparative effect of ΔN1 and ΔN2 mutations on gene expression, starting with the top DEGs in their respective PBS WT versus PBS mutant comparisons (Figures S15 and S16, Table S3, tabs 3.5 and 3.6). Among the 30 top DEGs in both comparisons, four (*Muc5.2*, *Prph2b*, *Gamt*, and *Opn1sw2*) are shared and all are upregulated (Figures S15 and S16). We also observed that several DEGs shared between the two mutant comparisons were immunity‐related (Table S3, tabs 3.5 and 3.6). The expression of some immunity‐related genes was validated using qPCR analysis targeting STAT1, IFI44a4, and ELF3 (Figure S19). In the LPS WT versus LPS mutant comparisons (Table S3, tabs 3.7 and 3.8), we found that three genes were shared among both comparisons (*si:ch73‐366l1.5*, *Npas4a*, and *Egr1*) (Figures S17 and S18). Expanding our analyses, we examined total DEGs in both PBS and LPS comparisons (Figure 6A,B, Table S3). We found that 2268 DEGs were shared between the two PBS comparisons, making up 59% and 51% of the total DEGs in the ΔN1 and ΔN2 comparisons, respectively (Figure 6A). 1103 DEGs were shared between the LPS comparisons, making up 21% and 69% of the total DEGs in the ΔN1 and ΔN2 comparisons, respectively (Figure 6B). GO analysis of these DEGs finds considerable similarity among the top dysregulated pathways, such as those related to nucleotide metabolism in the PBS comparisons and cell–cell adhesion in the LPS comparisons (Figure 6C,D). Interestingly, whether in the PBS or LPS conditions, axon guidance and neuron projection guidance appear among the top GO terms for WT versus mutant DEGs. We also observed an interesting phenomenon wherein numerous genes upregulated in PBS treated ΔN1 or ΔN2 lines compared to that found in PBS treated WT were found to be downregulated in LPS treated ΔN1 or ΔN2 lines compared to that found in LPS treated WT. We have annotated a select group of these genes, some of which are also immunity‐related (Figure 6E, Table S3). Collectively, these results demonstrate that the ΔN1 or ΔN2 lines have similar dysregulated gene expression.

rMATS was also conducted to examine DAS events in PBS or LPS WT versus ΔN1 or ΔN2 mutant conditions (Figure 6F,H, Table S4, tabs 4.9 and 4.10). We found that there were a total of 8338 and 7988 DAS events in the PBS ΔN1 or ΔN2 comparisons, respectively (Figure 6F). While there were a total of 8095 and 8080 DAS events in the LPS ΔN1 or ΔN2 comparisons, respectively (Figure 6H). We observed that 1087 genes were shared between DAS events and DEGs in the PBS WT versus PBS ΔN2 comparison and 427 genes in the LPS WT versus LPS ΔN2 comparison (Figure 6G,I). This would imply that 24% and 27% of the DEGs were due to alternative splicing in the PBS or LPS comparison, respectively. DAS event genes identified in the PBS or LPS WT versus ΔN2 comparisons were next analyzed for GO enrichment. Specifically, we evaluated these comparisons using the immunity‐related GO terms (Table S4, tab 4.8) employed in Figure 5C,E,G. Among the 18 immunity‐related GO terms that showed significant dysregulation in at least one comparison group, we found that only the response to bacterium GO term (GO:0009617) was shared between LPS WT versus LPS ΔN2 DEGs and DAS genes (Table S4, tab 4.11). This finding indicates that increased alternative splicing events in the ΔN2 line treated with LPS may contribute to the observed dysregulation of genes involved in the response to bacterium GO term. Other altered immunity‐related GO terms were, for the most part, specifically dysregulated in DEGs or DAS groups with little overlap (Table S4, tab 4.11).

### 3.8 Coilin N‐Terminal Mutant Hypomorph Alters the Innate Immune Response

**FIGURE 7:**
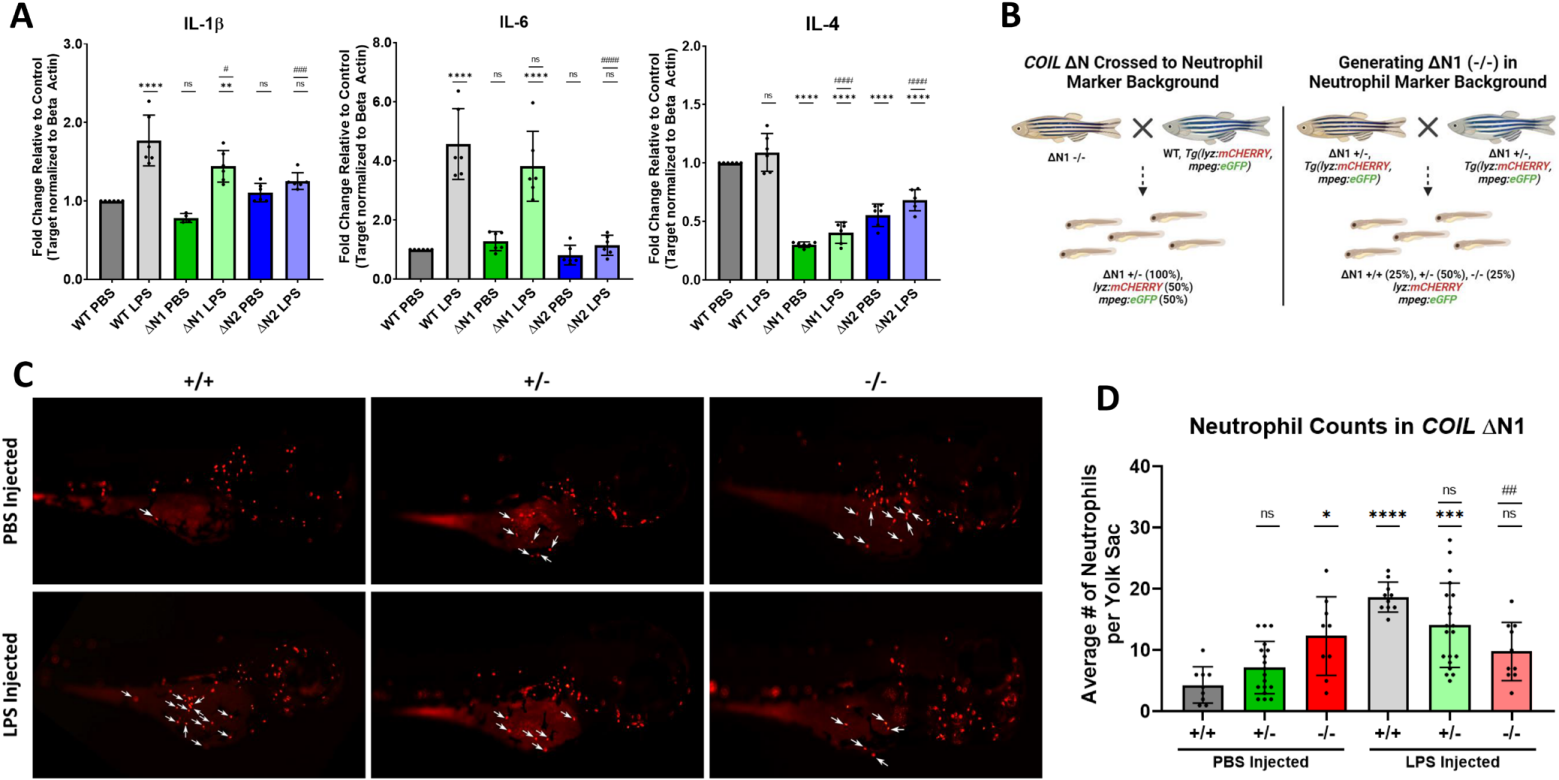
Coilin N‐terminal mutation impairs the immune response. (A) qPCR analysis of IL‐1β, IL‐6, and IL‐4 mRNA expression in 3 dpf WT or ΔN mutants injected with PBS or LPS. Data represents 3 biological replicates with 2 technical repeats for a total *N* = 6. Error bars represent SD and black dots represent individual data points. *p < 0.05, **p < 0.01, ***p < 0.001, ****p < 0.0001, ns = not significant for comparisons to WT PBS. ^#^ *p* < 0.05, ^##^ *p* < 0.01, ^###^ *p* < 0.001, ^####^ *p* < 0.0001 for comparisons to WT LPS. (B) Illustration of the generation of *COIL* ΔN zebrafish expressing the neutrophil marker (mCherry: *Lyz*) and macrophage marker (eGFP: *Mpeg*). (C) Representative images of neutrophil accumulation around the yolk sac following injection of PBS or LPS in 3 dpf *COIL* ΔN1 lineage zebrafish. Arrows indicate neutrophils at the yolk sac that were applied to counts. (+) indicates a normal *COIL* allele and (−) indicates a ΔN *COIL* allele. (D) Histogram displaying the average number of neutrophils per yolk sac following injection of PBS or LPS in 3 dpf *COIL* ΔN1 lineage zebrafish. Error bars represent SD and black dots represent individual data points. *p < 0.05, **p < 0.01, ***p < 0.001, ****p < 0.0001, ns = not significant for comparisons to (+/+) PBS. ^#^ *p* < 0.05, ^##^ *p* < 0.01, ^###^ *p* < 0.001, ^####^ *p* < 0.0001 for comparisons to (+/+) LPS.

To evaluate how coilin mutation might affect the immune response in zebrafish, we injected PBS or LPS into the yolk sac of 3 dpf embryos and collected 5 h post injection (hpi) for analysis. Our first analysis involved assessing the mRNA expression of pro‐inflammatory markers IL‐1β and IL‐6, as well as the anti‐inflammatory marker IL‐4, all of which have previously been found to be responsive to LPS injection in zebrafish [93]. Our results indicate that LPS injection was sufficient to induce a pro‐inflammatory response in WT embryos compared to PBS injected WT embryos (Figure 7A). However, IL‐4 did not appear to respond to LPS injection, whereas it was downregulated in a previous study [93] (Figure 7A). In both ΔN homozygous mutants, PBS injection did not alter the expression of pro‐inflammatory markers compared to WT PBS injected embryos, but interestingly, we observed an approximate 70% and 50% reduction of IL‐4 in PBS injected ΔN1 and ΔN2, respectively (Figure 7A). For IL‐1β, while both ΔN mutants exhibited an induction with LPS injection, we found that the degree of induction was significantly less than that observed in WT (Figure 7A). For IL‐6, we did not observe a difference between the inductions in LPS injected WT and ΔN1; however, there was a major blunting of IL‐6 induction in LPS injected ΔN2 (Figure 7A). For IL‐4, similar to the repression observed in PBS injected ΔN mutants, the anti‐inflammatory marker remained suppressed upon LPS injection (Figure 7A).

Next, we examined the activation of neutrophils and macrophages. We first crossed our *COIL* ΔN1 mutants into a transgenic background containing fluorescent markers for neutrophils *Tg*(*Lyz*:mCHERRY) and macrophages *Tg*(*Mpeg*:eGFP) (Figure 7B). At 3 dpf, these offspring were randomly divided into pools to receive PBS or LPS injection into the yolk sac. At 5 hpi, injected embryos were collected, fixed in paraformaldehyde, and their yolk sacs were imaged to assess the accumulation of neutrophils (Figure 7C) or macrophages (data not shown). Following imaging, neutrophils and macrophages were blindly counted for each embryo, and after counting was completed, embryos were processed to determine genotype. On average, we found that the number of neutrophils that accumulated in PBS injected embryos was 4.3 ± 2.9 in WTs (+/+), 7.2 ± 4.3 in hets (+/−), and 12.3 ± 6.4 in homozygotes (−/−) (Figure 7D). In LPS injected embryos, the average number of neutrophils was 18.7 ± 2.4 in WTs (+/+), 14.1 ± 6.9 in hets (+/−), and 9.8 ± 4.8 in homozygotes (−/−) (Figure 7D). We observed no effect on the accumulation of macrophages at the site of PBS or LPS injection between WTs, hets, or homozygous mutants (data not shown).

## 4 Discussion

### 4.1 Development of a Genetic Model of Coilin Deficiency in Zebrafish

Genetic models characterized by loss of function mutations in coilin have been generated in *Mus musculus* [89], *Drosophila melanogaster* [88], and *Arabidopsis thaliana* [23, 24, 94]. In all three models, coilin's mutation resulted in canonical CB abolishment, a finding that is supported by numerous cell culture studies [2, 95, 96]. Additionally, coilin KO in mouse reduces viability [89, 90], suggesting that coilin may be important in early development. This finding is also supported by cell culture studies indicating that coilin knockdown reduces cell proliferation [97, 98, 99], and a study of zebrafish embryos that resulted in embryonic lethality due to the suppression of coilin using morpholino injection [70]. However, it is important to note that there were no indications of reduced viability in the *D. melanogaster* or *A. thaliana* coilin KO models [23, 88, 94], and that the reduced viability in the mouse coilin KO study was observed only amongst the highly inbred mouse strains C57B1/6J and 129SvJ, with no effect on the outbred strain CD‐1 [90].

In this study, we have developed a novel model of coilin deficiency in zebrafish, characterized by the deletion of the first 26 aa and a 70% downregulation in protein expression. Based on the findings in previous genetic models, we had hypothesized that our N‐terminal coilin mutants would experience impaired CB assembly, particularly so because of a recent report showing that N‐terminal point mutations in human coilin abolish coilin self‐interaction and Nopp140 association [95]. However, we found no evidence of impaired CB assembly in our coilin mutants (Figure S3). Coilin provides the backbone on which the CB is assembled, but other proteins also contribute to CB formation, such as SMN [11], WRAP53 [9], and Nopp140 [95]. It is possible that these other major CB components can facilitate CB assembly even with a reduced level of full‐length coilin. We also find no evidence of reduced viability amongst our coilin‐deficient mutants. Again, this may be due to the presence of mostly full‐length coilin in these embryos that allows them to develop without issue, compared to the morpholino injected embryos and the inbred mouse KOs. Alternatively, it could be attributed to the intrastrain genetic variation that is common amongst inbred zebrafish strains [100], allowing for protection against the reduced coilin levels similar to the outbred mouse KOs.

### 4.2 A Role for Vertebrate Coilin in the Immune Response

In plants, coilin positively contributes to the regulation of genes involved in innate immunity [23, 30, 94, 101, 102, 103]. Alternative splicing has been found to be an important tool in regulating gene expression and activity of immune cells belonging to both the innate and adaptive response [104]. As coilin and CBs are known to facilitate the maturation of spliceosome [7, 12, 15, 16, 17], we wanted to determine if impaired splicing activity due to coilin suppression in HFF cells or mutation in zebrafish would influence the immune response. In this study, we show that coilin KD in HFF cells or mutation in zebrafish embryos results in a substantial increase in differential alternative splicing events. However, we find little evidence of an overlap between the genes affected by DAS events and immunity‐related genes that are differentially expressed, especially in the HFF model. This would suggest that coilin's role in splicing is not the sole method by which coilin impacts the immune response. These results are consistent with a study of plant coilin in which the authors compared the transcriptomes of a WT and coilin mutant plant with a particular focus on immunity‐related genes [23]. When considered together, these findings implicate a novel function for coilin apart from its canonical role in CB function. This novel function may involve the post‐translational modification of immunity pathway proteins by SUMOylation [32, 34].

## 5 Conclusions

These experiments highlight an important role for coilin as a regulator of the innate immune response in vertebrate animals. In human cells lacking many CBs, we have shown that coilin KD under DMSO or LPS conditions suppresses the expression of immunity‐related genes. This includes the dysregulated expression of certain miRNAs thought to regulate immune pathways. We have also shown that a CRISPR/Cas9 engineered mutation in zebrafish, producing a coilin‐deficient phenotype, results in the impaired expression of inflammation markers and dysregulated activation of neutrophils under PBS or LPS injection. Likewise, we find that the activation of immunity‐related pathways is suppressed in coilin mutants under LPS injection and the expression of several immunity‐related genes is induced under PBS injection. Collectively, we present strong evidence of a functional role for vertebrate coilin in regulating the immune response.

## Author Contributions

Douglas M. McLaurin designed research, performed research, analyzed data, and drafted and edited the manuscript; Sara K. Tucker performed research, analyzed data, and edited the manuscript; Shanzida J. Siddique analyzed data, conducted bioinformatics analysis, and edited the manuscript; Lavanya Challagundla analyzed data, conducted bioinformatics analysis, and edited the manuscript; Yann Gibert designed research, analyzed data, and drafted and edited the manuscript; Michael D. Hebert designed research, performed research, analyzed data, and drafted and edited the manuscript.

## Conflicts of Interest

The authors declare no conflicts of interest.

## Supporting information


Table S1.

Table S2.

Table S3.

Table S4.

Figure S1.

## Data Availability

The raw and processed RNA seq data from human foreskin fibroblast cell experiments can be accessed from the Gene Expression Omnibus (GEO) via accession GSE268257. Small RNA seq data from HFF cell experiments have been deposited at NCBI GEO accession GSE271895. The raw and processed RNA seq data from zebrafish experiments can be accessed from the GEO via accession GSE273717.
