# Supplementary material for: A Novel Role for Coilin in Vertebrate Innate Immunity
Source: FASEB J. 2025 Apr 25;39(8):e70580. doi: 10.1096/fj.202403276R (PMC12023821; doi:10.1096/fj.202403276R)
Supplement: Supplementary file 5 — Figure S1. [file FSB2-39-e70580-s001.pdf]

Experiment	Ensembl ID	Gene	baseMean	log2FoldChange	lfcSE	stat	pvalue	padj
N DMSO vs I2 DMSO	ENSG00000121058	*COIL*	823.8422	-2.32632	0.147337	-15.789	3.70E-56	3.43E-53
N LPS vs I2 LPS	ENSG00000121058	*COIL*	823.8422	-2.05493	0.144612	-14.21	7.95E-46	1.05E-42
N DMSO vs IA DMSO	ENSG00000121058	*COIL*	823.8422	-2.17855	0.145641	-14.9583	1.37E-50	1.92E-47
N LPS vs IA LPS	ENSG00000121058	*COIL*	823.8422	-1.82272	0.14389	-12.6675	8.95E-37	9.33E-34
N DMSO vs W DMSO	ENSG00000141499	*WRAP53*	920.534	-2.55232	0.160936	-15.8592	1.21E-56	1.96E-52
N LPS vs W LPS	ENSG00000141499	*WRAP53*	920.534	-2.6799	0.16327	-16.4139	1.52E-60	1.29E-56

Figure S1. DEG analysis results from HFF RNAseq under various comparisons. This table illustrates the downregulation of coilin under coilin KD when compared to control KD and the downregulation of WRAP53 under WRAP53 KD when compared to control KD.

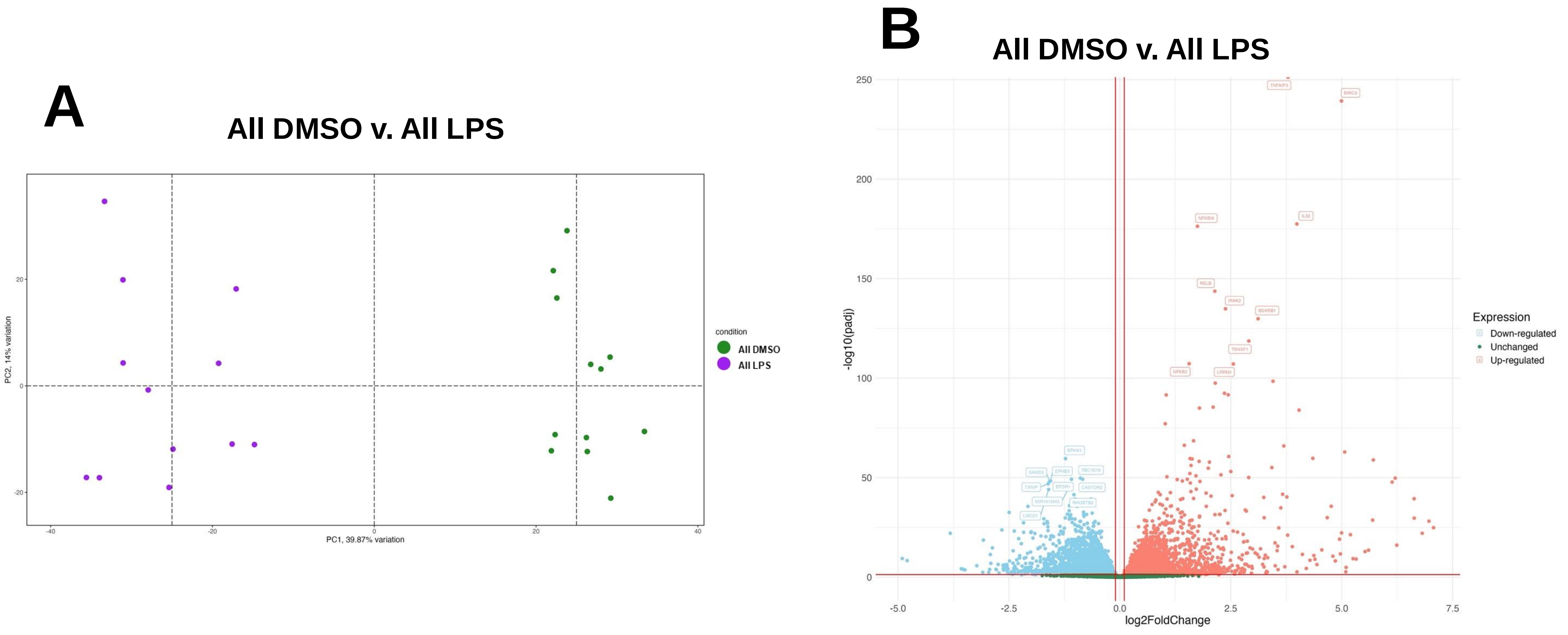

Figure S2. A) PCA plot displaying similarities between DMSO and LPS treated conditions. B) Volcano plot of DMSO and LPS treated conditions.

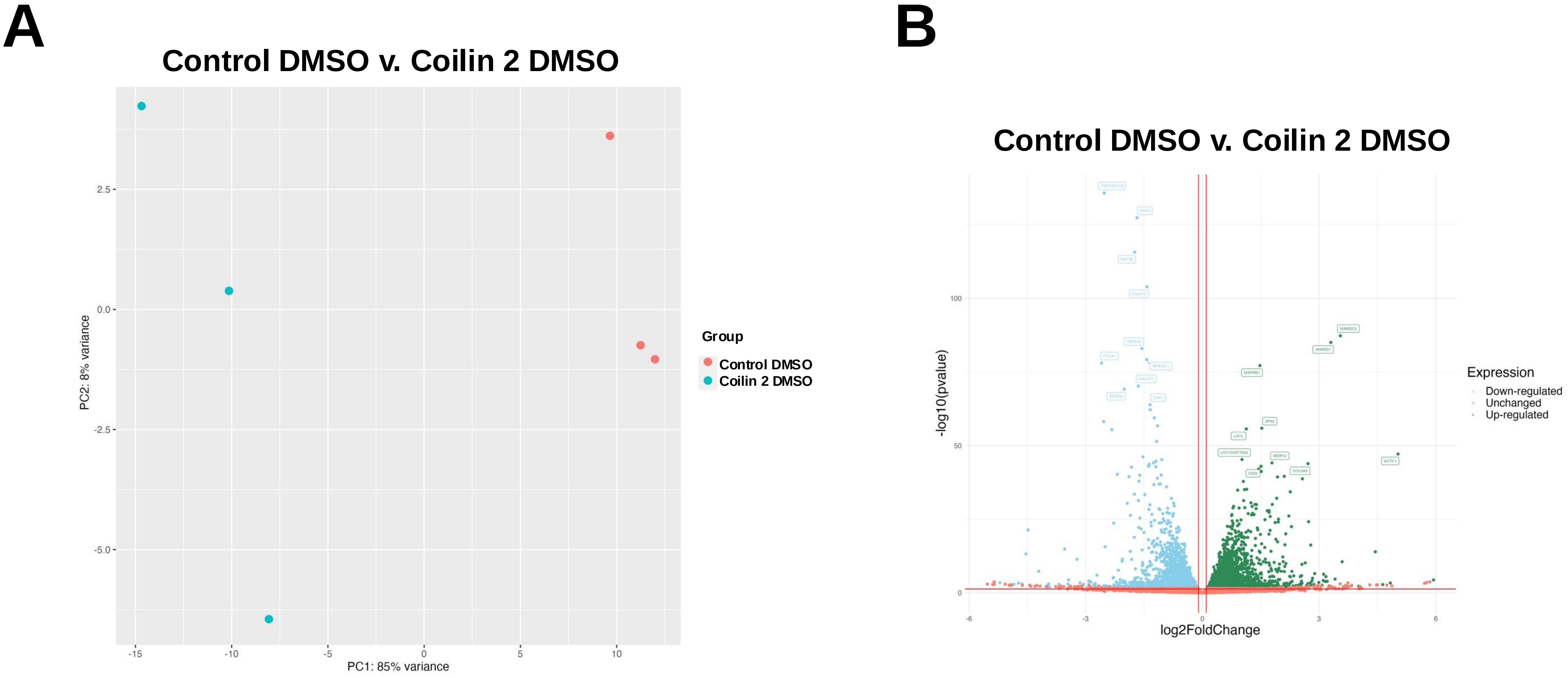

Figure S3. A) PCA plot displaying similarities between control (N) and coilin 2 (I2) knockdowns in untreated conditions. B) Volcano plot of control (N) and coilin 2 (I2) knockdowns in untreated conditions.

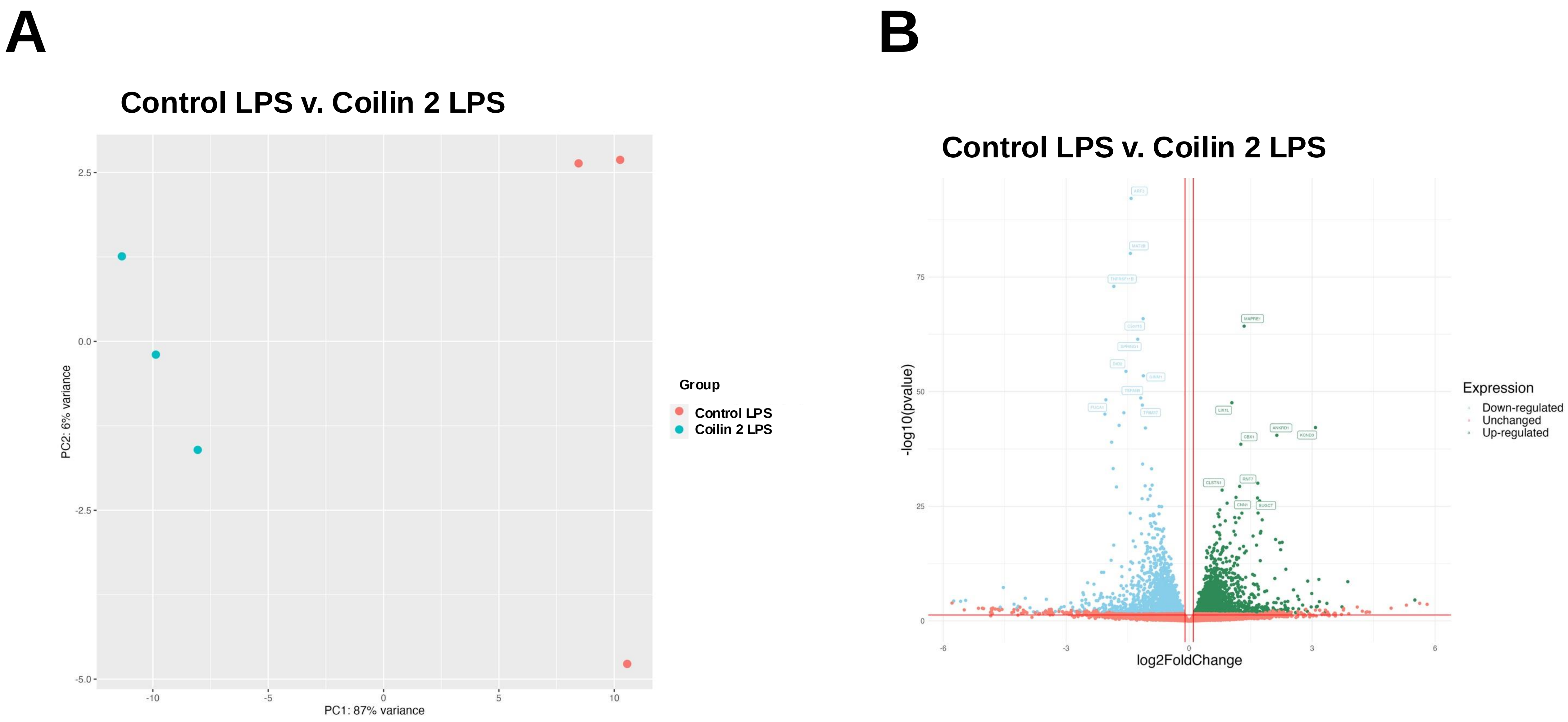

Figure S4. A) PCA plot displaying similarities between control (N) and coilin 2 (I2) knockdowns in LPS treated conditions. B) Volcano plot of control (N) and coilin 2 (I2) knockdowns in LPS treated conditions.

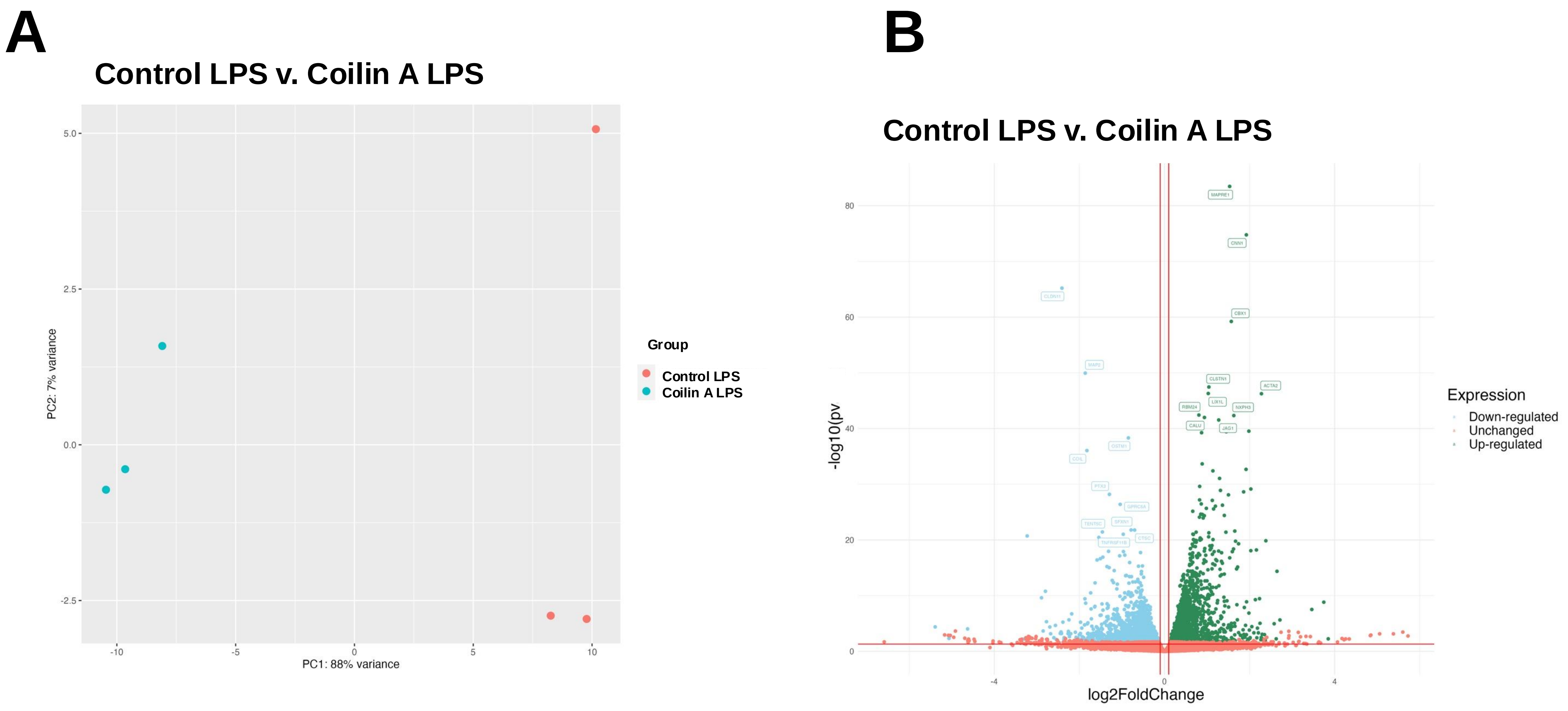

Figure S5. A) PCA plot displaying similarities between control (N) and coilin A (IA) knockdowns in LPS treated conditions. B) Volcano plot of control (N) and coilin A (IA) knockdowns in LPS treated conditions.

A

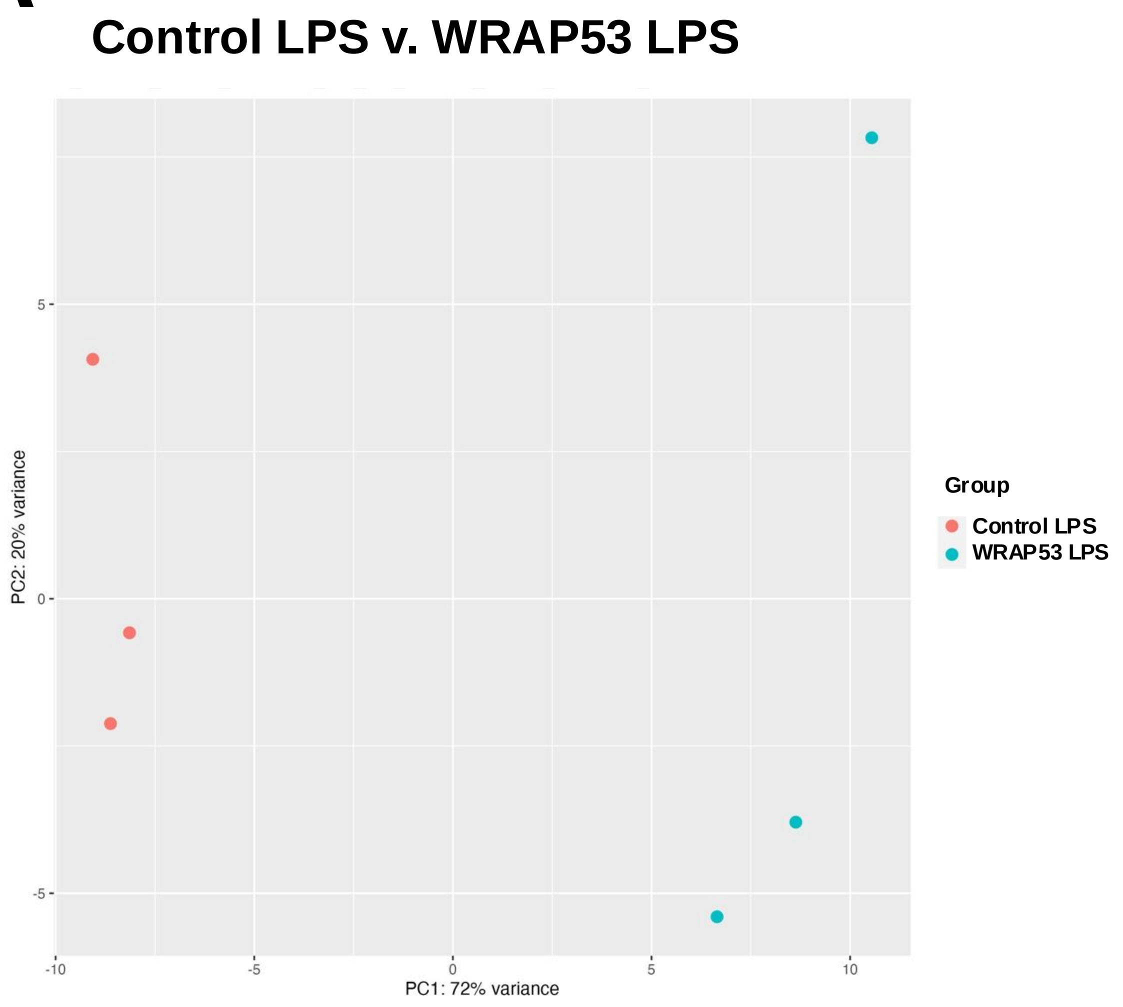

B

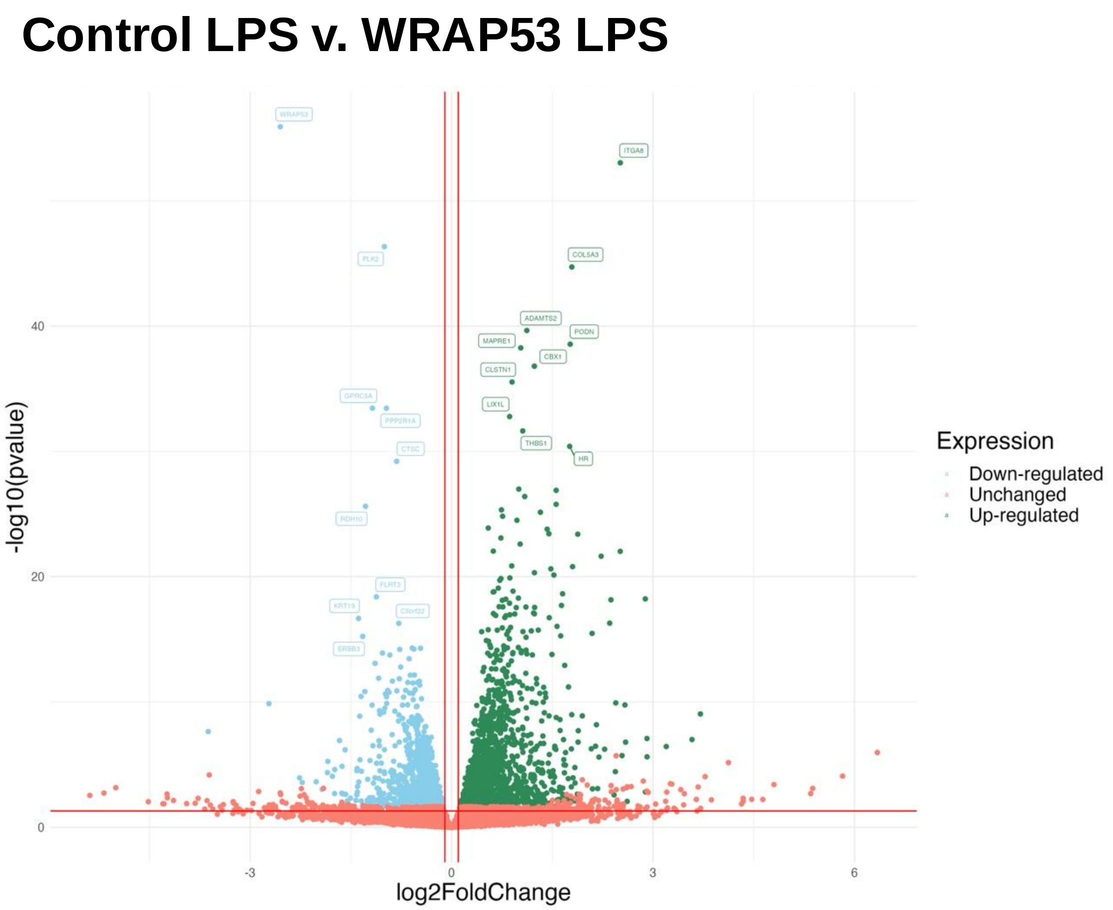

Figure S6. A) PCA plot displaying similarities between control (N) and WRAP53 (W) knockdowns in LPS treated conditions. B) Volcano plot of control (N) and WRAP53 (W) knockdowns in LPS treated conditions.

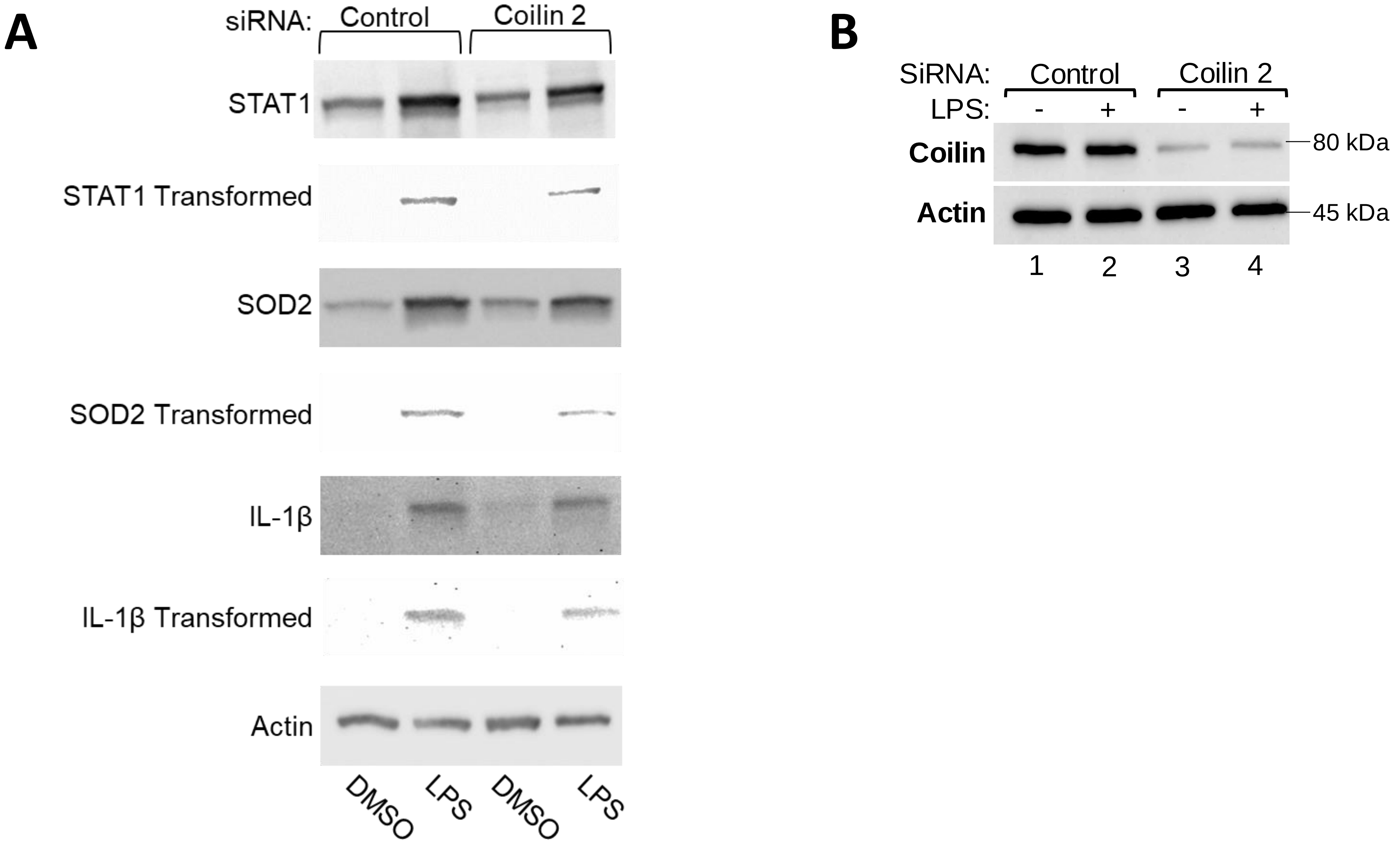

Figure S7. A) HFF cells transfected with either control or coilin 2 siRNA. Each siRNA transfection was also treated with either LPS (2 µg/mL) or DMSO as indicated. Protein was collected and subject to Western blot analysis with antibodies to STAT1, SOD2, and IL-1β. Images were transformed by adjusting exposures over the entire image and are shown here for ease of visualization. Actin probing is shown for total protein amount. B) Coilin probing of cell lysate used in A showing reduction of coilin protein upon coilin siRNA treatment.

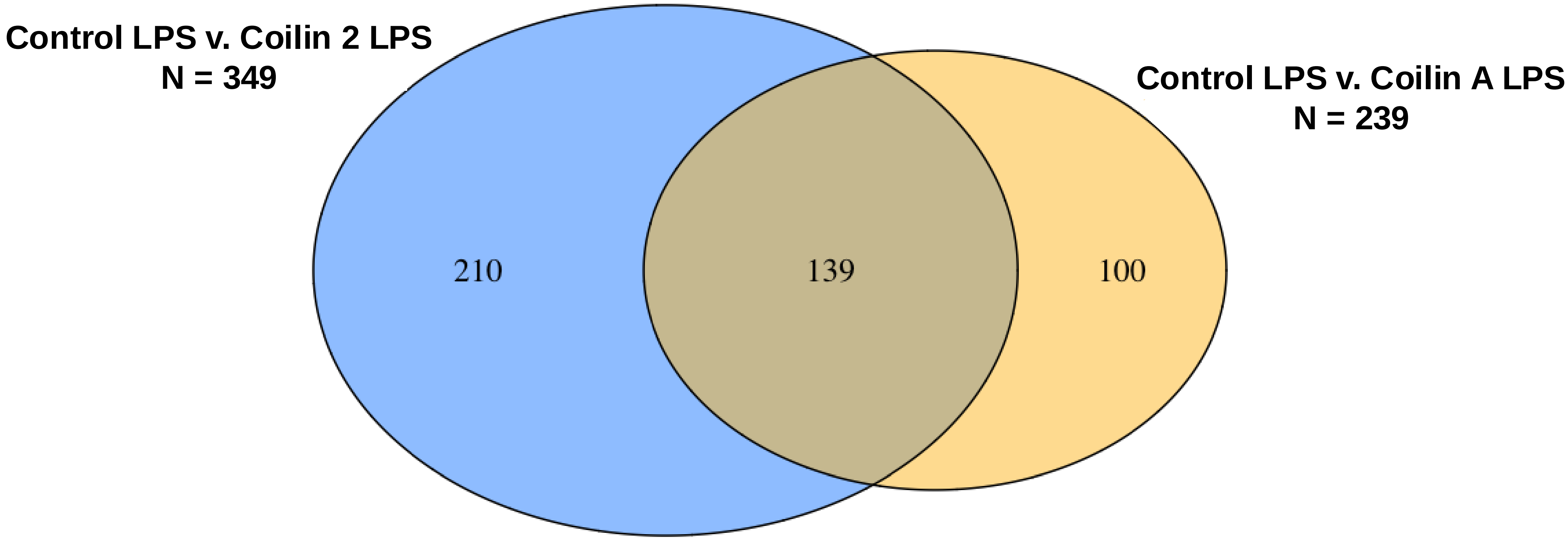

Figure S8. Venn diagram illustrating the overlap in differentially expressed immunity-related genes that are enriched in the GO terms found in Figure 1D and 1F.

WT

```
  1 ATG GCC ACC TCC AGC CTC AAC ACT GCC AGG GTT AGG TTG TAT TTT GAC TAT CCT CCT CCT GCA ACA CCA GAG TGT CGG ATG TGT TGG TTG
  1  M   A   T   S   S   L   N   T   A   R   V   R   L   Y   F   D   Y   P   P   P   A   T   P   E   C   R   M   C   W   L

 91 CTG GTG GAC CTG AAC AAA TGC CGA GTT GTC GCA GAT CTT TCC AGC ATC ATA AAG GAA AAG TTT GGC TAC AGC CGC AAG ACT ATT TTA GAC
 31  L   V   D   L   N   K   C   R   V   V   A   D   L   S   S   I   I   K   E   K   F   G   Y   S   R   K   T   I   L   D

181 CTG TTT ATT GAG GAA TGC TAC CTG CCT TCT GCT GAA AGT ATT TAT ATA GTA CGT GAT AAT GAC AGT GTA
 61  L   F   I   E   E   C   Y   L   P   S   A   E   S   I   Y   I   V   R   D   N   D   S   V
```

ΔN1

```
  1 ATG GCC ACC TCC AGC CTC AAC ACT GCC AGG GTT AGG TTG TAT TTT GAC TAT CCT CCT CCT GCA ACA CCT GTC GGA TGT GTT GGT TGC TGG
  1  M   A   T   S   S   L   N   T   A   R   V   R   L   Y   F   D   Y   P   P   P   A   T   P   V   G   C   V   G   C   W

 91 TGG ACC TGA
 31  W   T   *
```

ΔN2

```
  1 ATG GCC ACC TCC AGC CTC AAC ACT GCC AGG GTT AGG TTG TAT TTT GAC TAT CCT CCT CCT GCA ACA CCA GAT GTG TTG GTT GCT GGT GGA
  1  M   A   T   S   S   L   N   T   A   R   V   R   L   Y   F   D   Y   P   P   P   A   T   P   D   V   L   V   A   G   G

 91 CCT GAA CAA ATG CCG AGT TGT CGC AGA TCT TTC CAG CAT CAT AAA GGA AAA GTT TGG CTA CAG CCG CAA GAC TAT TTT AGA CCT GTT TAT
 31  P   E   Q   M   P   S   C   R   R   S   F   Q   H   H   K   G   K   V   W   L   Q   P   Q   D   Y   F   R   P   V   Y

181 TGA
 61  *
```

Figure S9. Predicted amino acid sequences of N-terminal coilin mutants. Red highlights represent non-native coilin amino acid sequence. * represents stop codon.

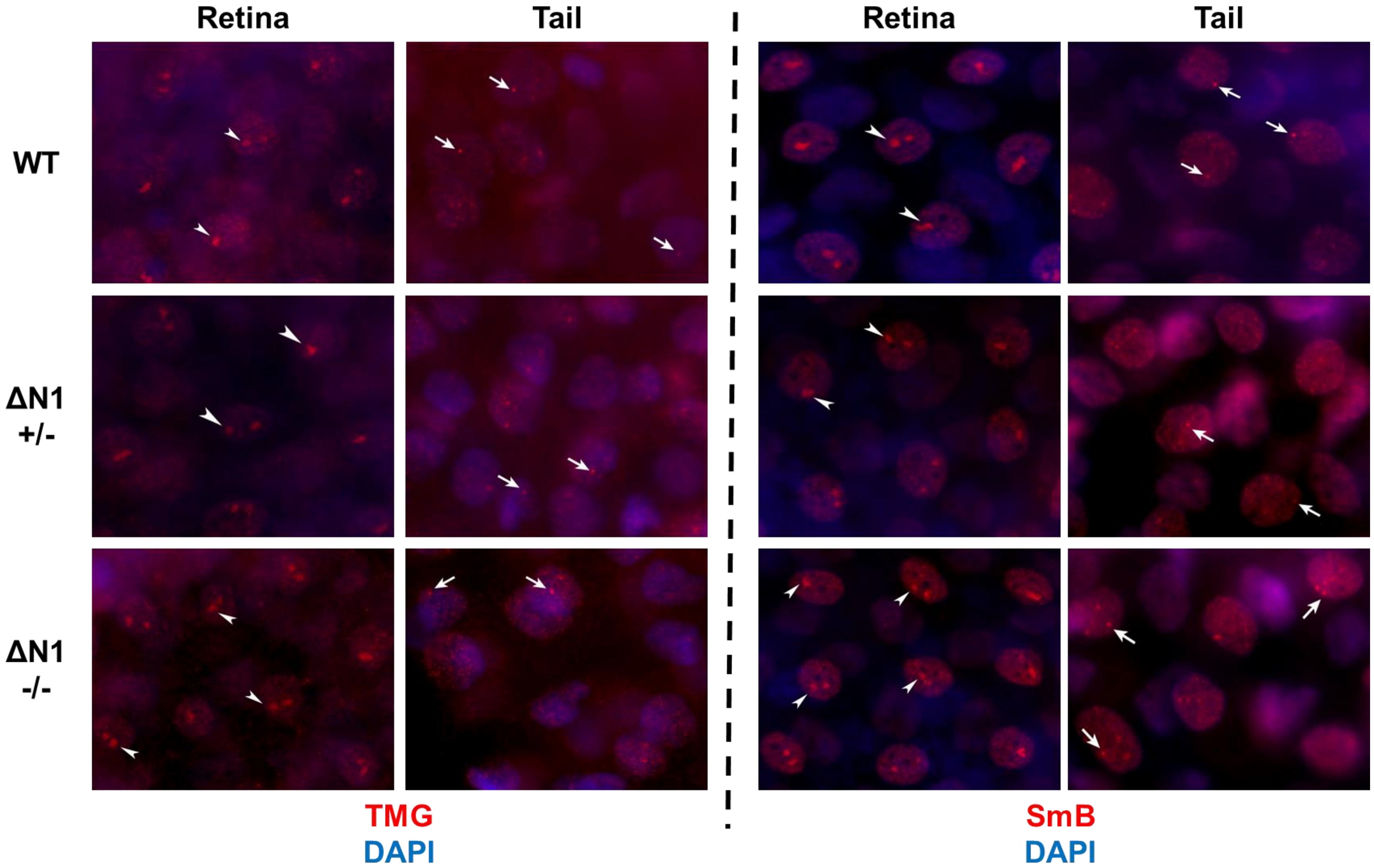

Figure S10. 48 hpf WT, ΔN1 heterozygous (+/-), or ΔN1 homozygous (-/-) zebrafish embryos probed with (TMG) or (SmB). Images of nuclear foci and presumptive CBs were taken in the retina or in the tail region toward the caudal fin. Arrowheads indicated nuclear foci observed in the retina and arrows indicate presumptive CBs in the tail region.

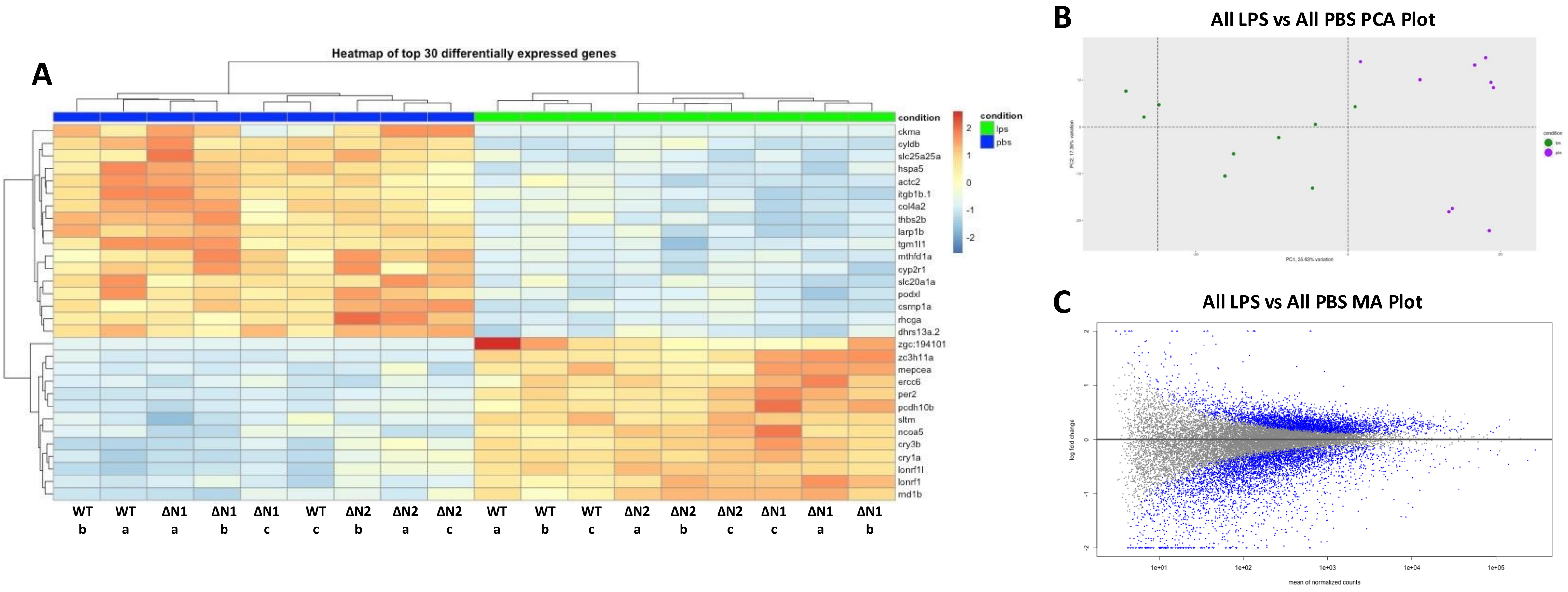

Figure S11. Heatmap of the top 30 differentially expressed genes for WT and mutant embryos injected with PBS or LPS. B) Principal component analysis (PCA) plot displaying the similarities between WT and mutant embryos injected with PBS or LPS. C) MA plot of WT and mutant embryos injected with PBS or LPS.

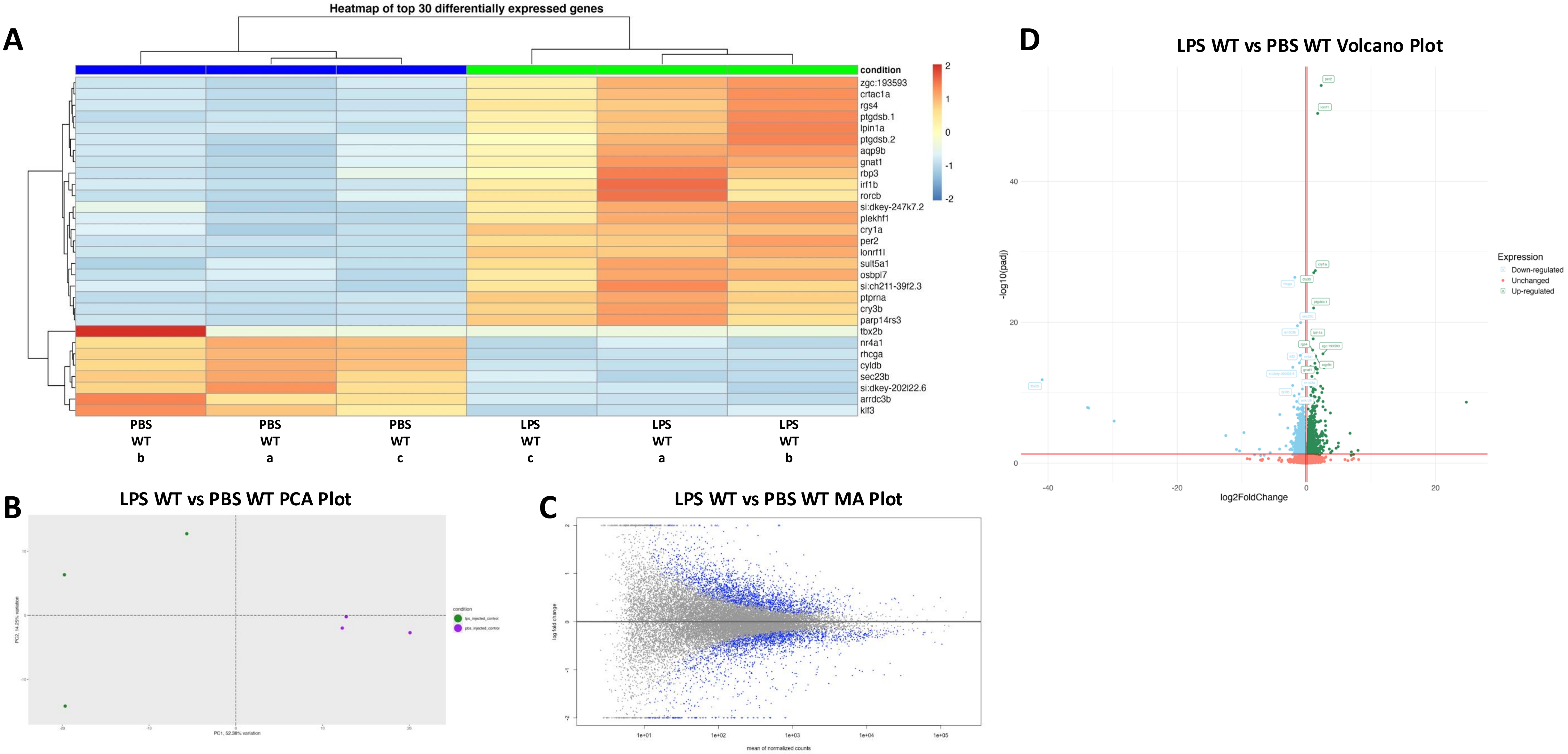

Figure S12. Heatmap of the top 30 differentially expressed genes for WT embryos injected with PBS or LPS. B) Principal component analysis (PCA) plot displaying the similarities between WT embryos injected with PBS or LPS. C) MA plot of WT embryos injected with PBS or LPS. D) Volcano plot of WT embryos injected with PBS or LPS.

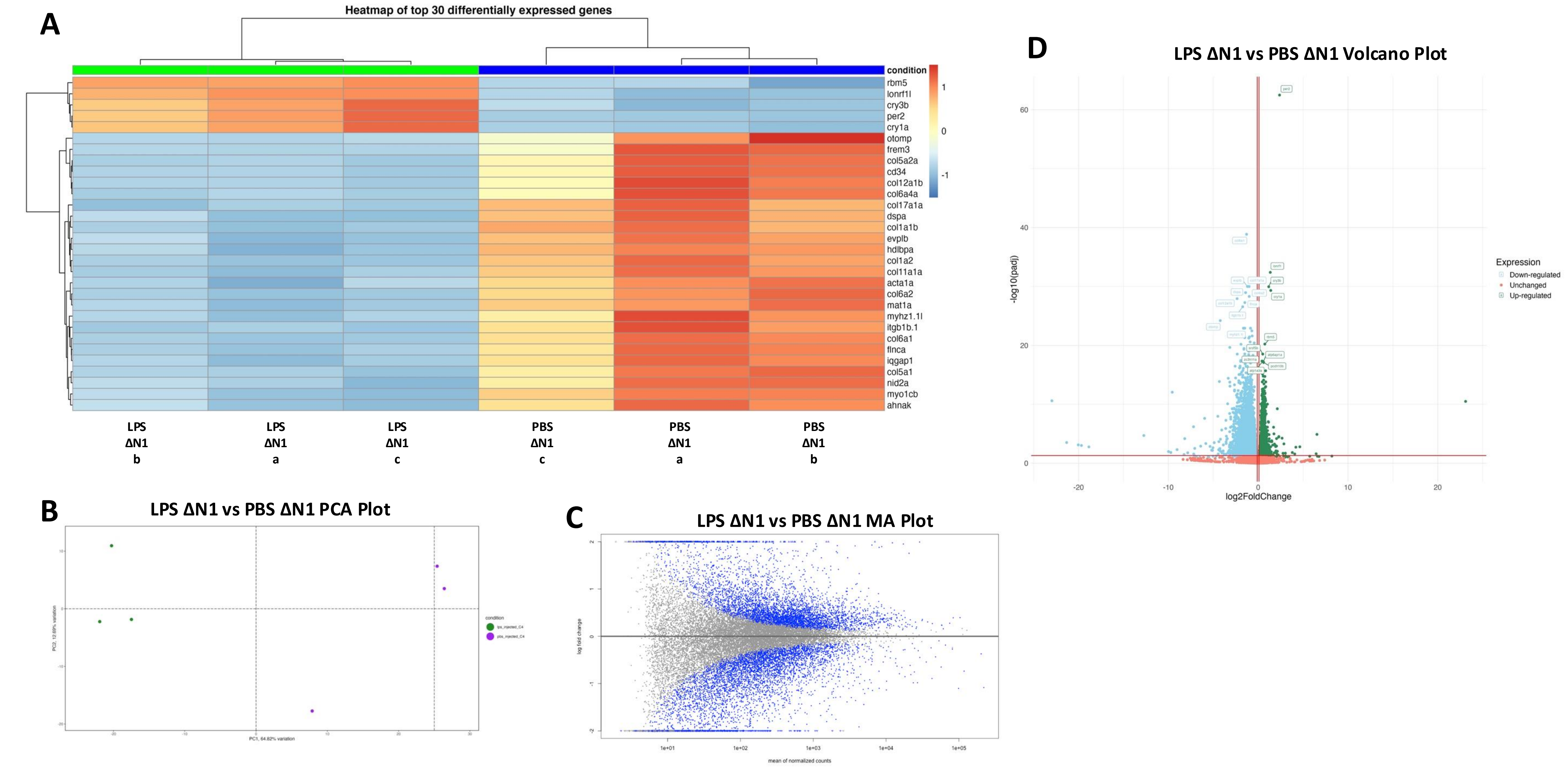

Figure S13. Heatmap of the top 30 differentially expressed genes for ΔN1 mutant embryos injected with PBS or LPS. B) Principal component analysis (PCA) plot displaying the similarities between ΔN1 mutant embryos injected with PBS or LPS. C) MA plot of ΔN1 mutant embryos injected with PBS or LPS. D) Volcano plot of ΔN1 mutant embryos injected with PBS or LPS.

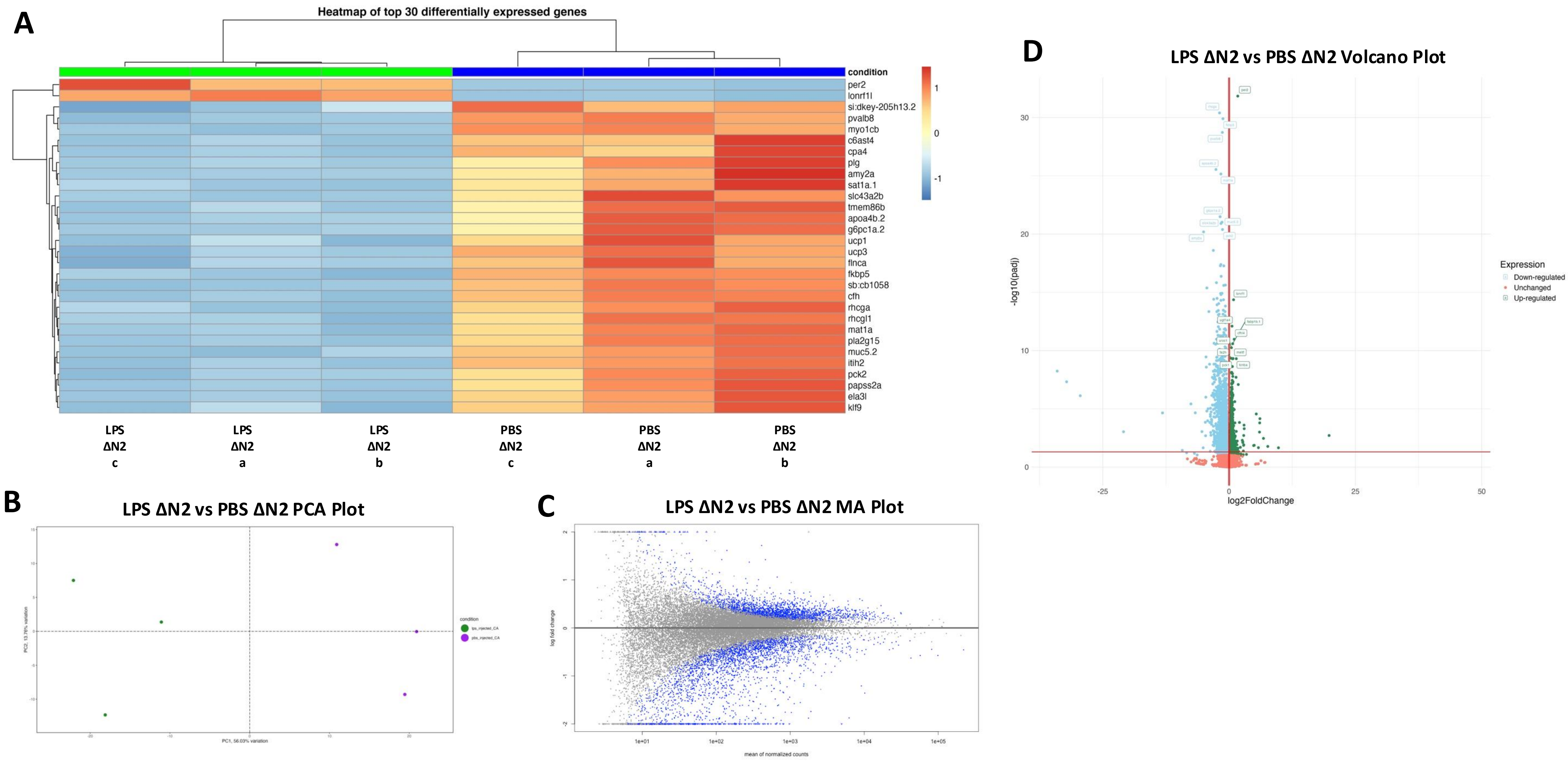

Figure S14. Heatmap of the top 30 differentially expressed genes for ΔN2 mutant embryos injected with PBS or LPS. B) Principal component analysis (PCA) plot displaying the similarities between ΔN2 mutant embryos injected with PBS or LPS. C) MA plot of ΔN2 mutant embryos injected with PBS or LPS. D) Volcano plot of ΔN2 mutant embryos injected with PBS or LPS.

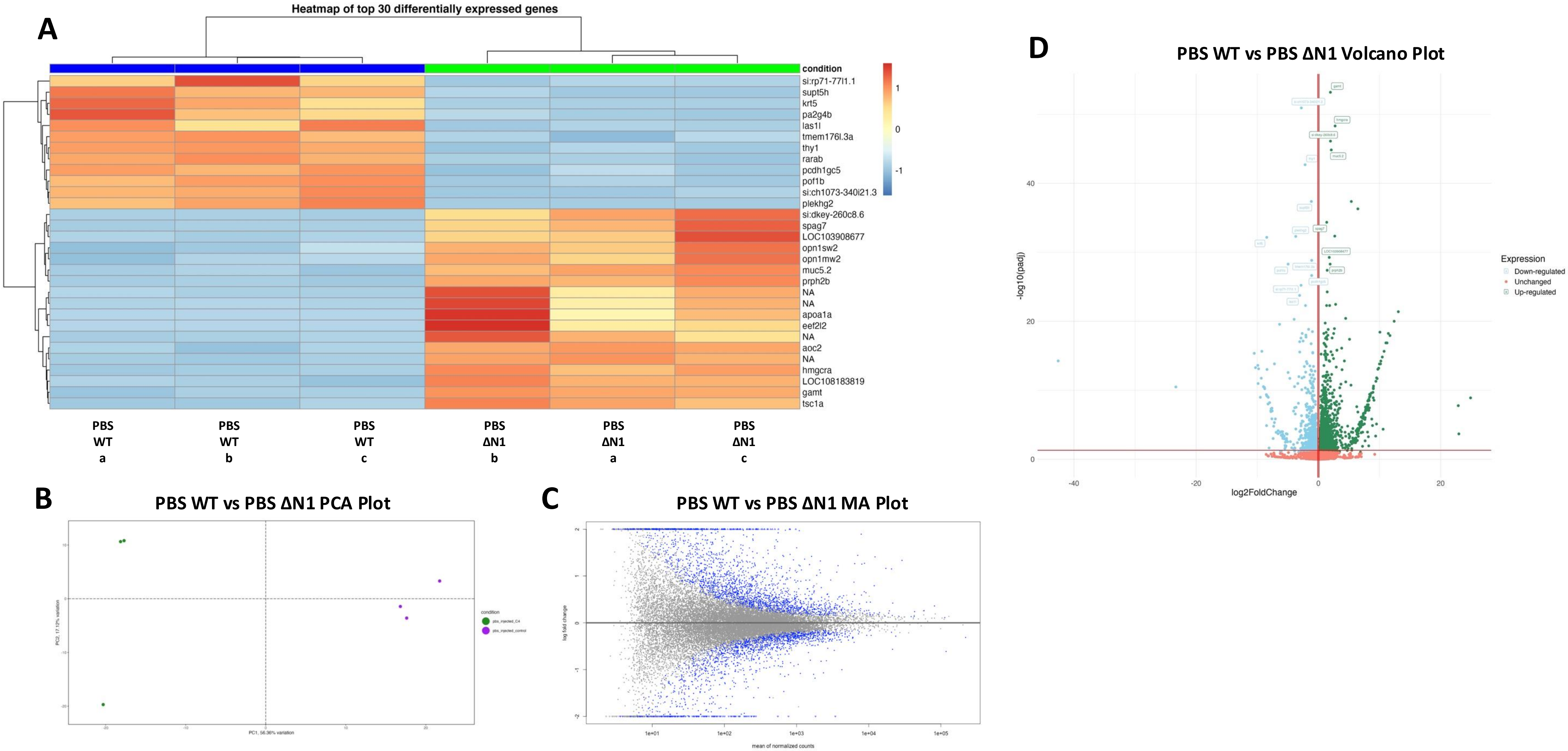

Figure S15. Heatmap of the top 30 differentially expressed genes for WT and ΔN1 mutant embryos injected with PBS. B) Principal component analysis (PCA) plot displaying the similarities between WT and ΔN1 mutant embryos injected with PBS. C) MA plot of WT and ΔN1 mutant embryos injected with PBS. D) Volcano plot of WT and ΔN1 mutant embryos injected with PBS.

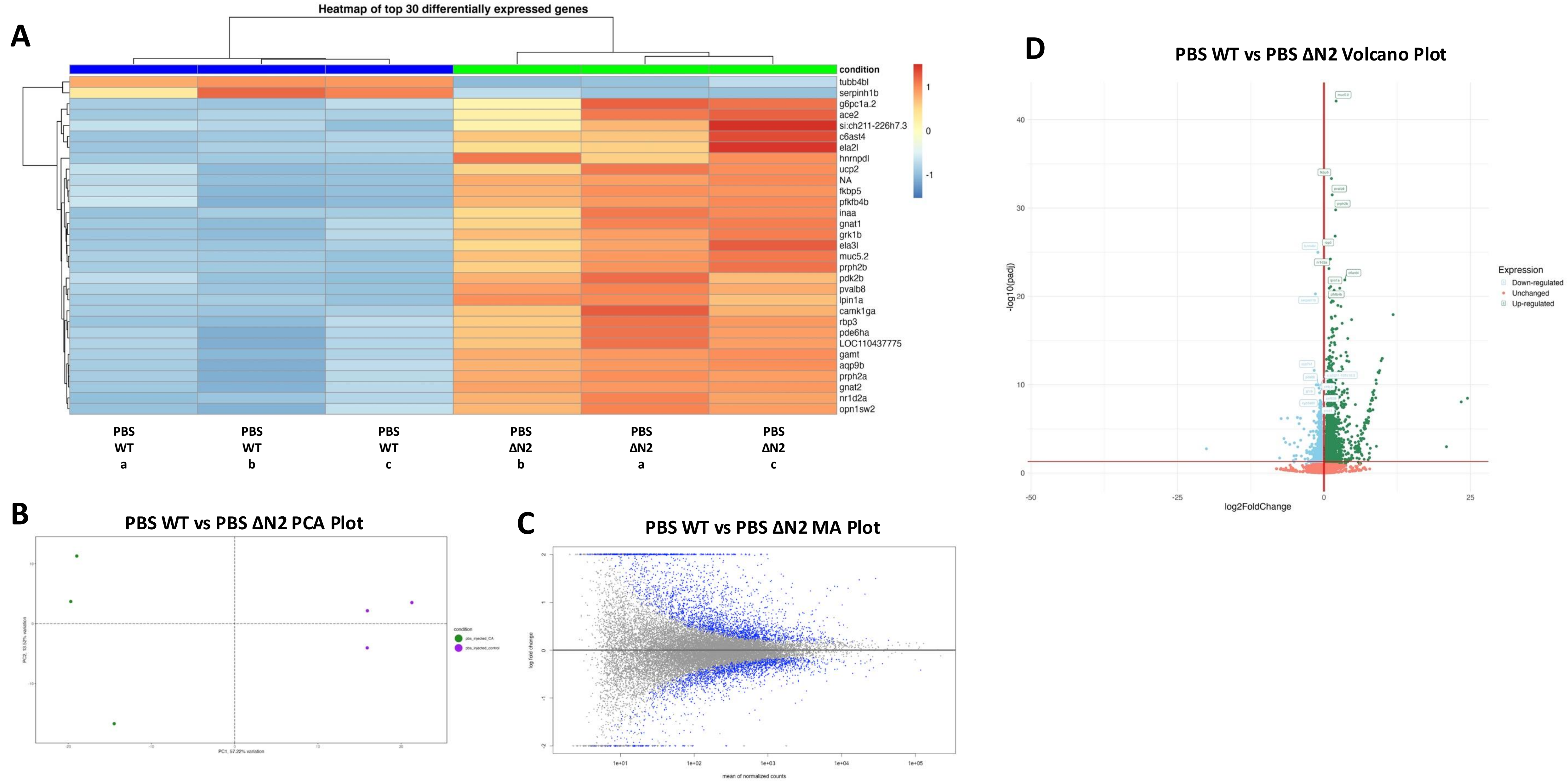

Figure S16. Heatmap of the top 30 differentially expressed genes for WT and ΔN2 mutant embryos injected with PBS. B) Principal component analysis (PCA) plot displaying the similarities between WT and ΔN2 mutant embryos injected with PBS. C) MA plot of WT and ΔN2 mutant embryos injected with PBS. D) Volcano plot of WT and ΔN2 mutant embryos injected with PBS.

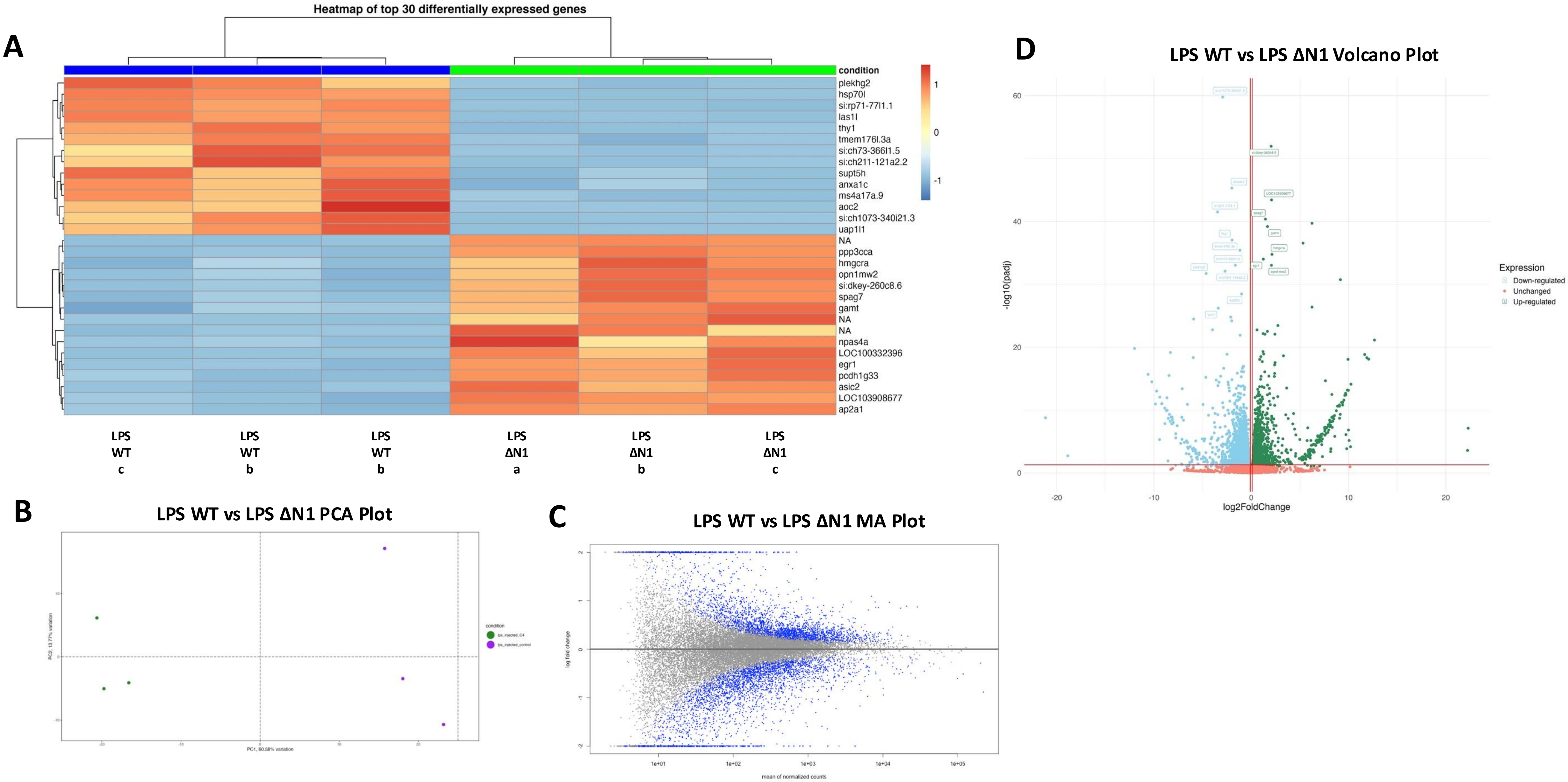

Figure S17. Heatmap of the top 30 differentially expressed genes for WT and ΔN1 mutant embryos injected with LPS. B) Principal component analysis (PCA) plot displaying the similarities between WT and ΔN1 mutant embryos injected with LPS. C) MA plot of WT and ΔN1 mutant embryos injected with LPS. D) Volcano plot of WT and ΔN1 mutant embryos injected with LPS.

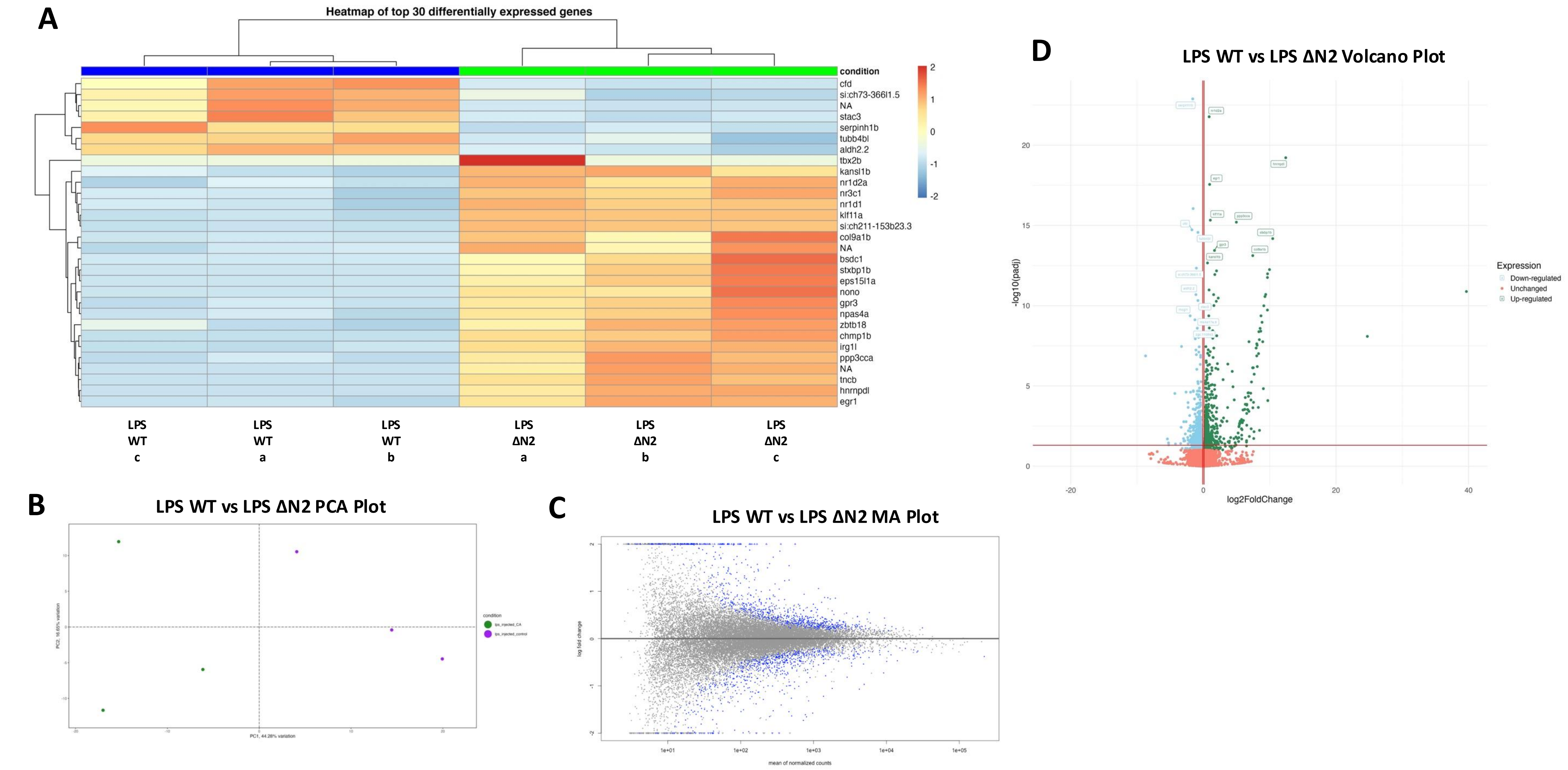

Figure S18. Heatmap of the top 30 differentially expressed genes for WT and ΔN2 mutant embryos injected with LPS. B) Principal component analysis (PCA) plot displaying the similarities between WT and ΔN2 mutant embryos injected with LPS. C) MA plot of WT and ΔN2 mutant embryos injected with LPS. D) Volcano plot of WT and ΔN2 mutant embryos injected with LPS

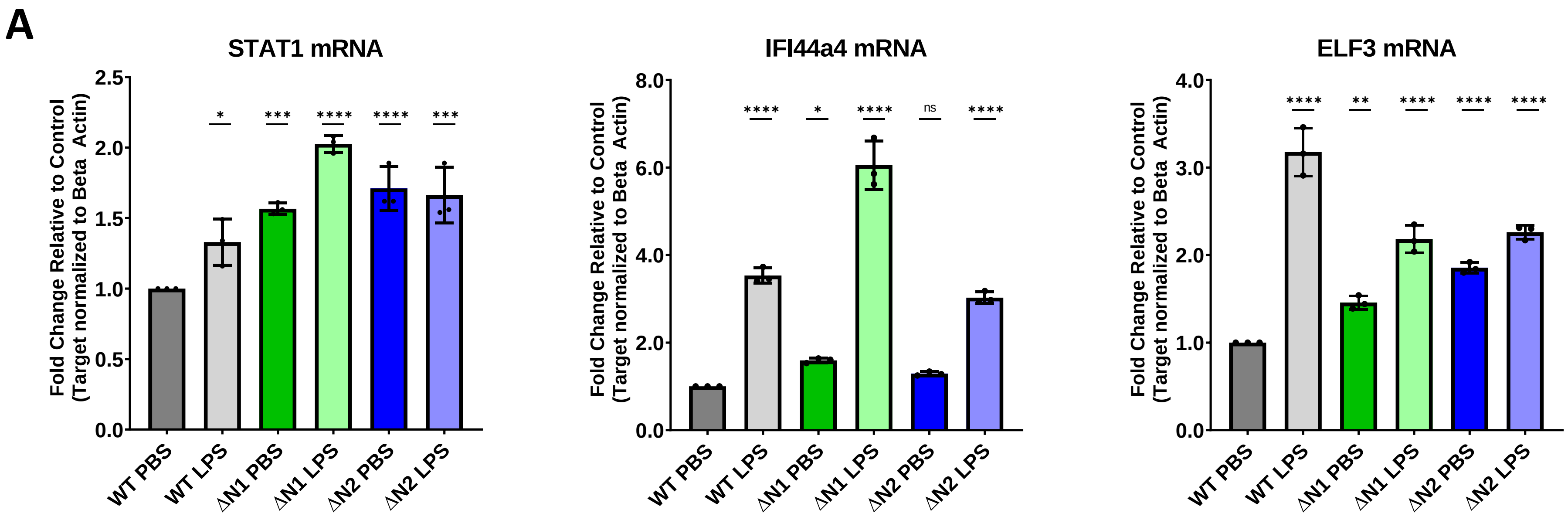

B

Salmon Counts from RNA Samples Used for Validation

Gene Name	PBS WT	LPS WT	PBS ΔN1	LPS ΔN1	PBS ΔN2	LPS ΔN2
STAT1a	400.6196	622.9933	583.5107	581.2272	654.5484	561.1733
IFI44a4	19.90042	110.76	38.33879	172.7593	31.49116	106.9923
ELF3	210.5644	635.1819	288.9359	391.548	362.8035	461.7167

Figure S19. A) qPCR analysis to validate the expression of targets in 3 dpf WT or ΔN double mutants injected with PBS or LPS. N = 3, error bars represent SD and black dots represent individual data points. * = $p < 0.05$, ** = $p < 0.01$, *** = $p < 0.001$, **** = $p < 0.0001$, ns = not significant for comparisons to WT PBS. B) Table displaying the counts of target genes in the RNA samples used for validation qPCR.
